# DECM: A Discrete Element for Multiscale Modeling of Composite Materials Using the Cell Method

**DOI:** 10.3390/ma13040880

**Published:** 2020-02-16

**Authors:** Elena Ferretti

**Affiliations:** Department of Civil, Environmental and Materials Engineering—DICAM, Alma Mater Studiorum Università di Bologna, 40136 Bologna, Italy; elena.ferretti2@unibo.it; Tel.: +39-051-209-35-15

**Keywords:** discrete element method, cell method, multiscale modeling, periodic composite materials, nonlocality

## Abstract

This paper presents a new numerical method for multiscale modeling of composite materials. The new numerical model, called DECM, consists of a DEM (Discrete Element Method) approach of the Cell Method (CM) and combines the main features of both the DEM and the CM. In particular, it offers the same degree of detail as the CM, on the microscale, and manages the discrete elements individually such as the DEM—allowing finite displacements and rotations—on the macroscale. Moreover, the DECM is able to activate crack propagation until complete detachment and automatically recognizes new contacts. Unlike other DEM approaches for modeling failure mechanisms in continuous media, the DECM does not require prior knowledge of the failure position. Furthermore, the DECM solves the problems in the space domain directly. Therefore, it does not require any dynamic relaxation techniques to obtain the static solution. For the sake of example, the paper shows the results offered by the DECM for axial and shear loading of a composite two-dimensional domain with periodic round inclusions. The paper also offers some insights into how the inclusions modify the stress field in composite continua.

## 1. Introduction

The most commonly used numerical techniques for modeling the behavior of composite materials make use of the Finite Element Method (FEM) [1]. The main limitation of these computational methods is to describe the problem on a macro-scale or meso-scale, idealizing the material as a continuum with some homogenization technique [2,3]. Therefore, they are not useful for modeling phenomena up to the scale of single inclusions or interfaces between sub-domains. In particular, although it is possible to introduce a discontinuity if known in advance, they cannot simulate the distinct property of the discontinuity, which is necessary to describe the large displacements of particles in geomaterials.

The Cell Method (CM) is a direct algebraic method [4] that uses global variables instead of the FEM field variables. Since the global variables involved in obtaining the direct algebraic formulation does not need to be differentiable functions, the range of applicability of the algebraic formulation has no restrictions, while the differential formulation used in FEM models is restricted to regions without material discontinuities or concentrated sources. In fact, the global variables are continuous across the interface of two different media, while their variations—hence, the field variables—can be discontinuous [5]. This allows the CM to model domains made up of different materials without requiring any homogenization technique [6,7,8].

Unlike the FEM and the CM, the Discrete Element Method (DEM)—also called the Distinct Elements Method—models the materials as an assembly of separate particles, which allows simulation not only of geomaterials, but also of any particulate matter, such as powders or granules [9]. The particles (rigid or deformable [10]) move according to Newton’s second law of motion and interact by contact constitutive laws (rigid or deformable). The simulation of geo-mechanical problems is rather simple with a DEM approach and the results are very accurate, even though they may require too much computation time for current computer technology [11]. Also for this reason, the range of applicability of the DEM in its early formulation [12]—derived from Cundall’s studies on discontinuum mechanics [13]—is limited to the micro-scale.

Although the main task of the DEM is to model discontinuities, it is also possible to use a DEM code to model the continuum [14,15,16,17,18,19], through particular contact constitutive relations. In the specific case of materials consisting of a matrix that cements aggregates not in direct contact, these constitutive relations establish interactions between the aggregates when they are within an interaction range greater than the distance between the centroids of the aggregates [18]. This allows studying damage in homogeneous materials such as ceramics [20] and heterogeneous materials such as concrete [21,22,23], rock [24], or composites with a brick and mortar architecture (both natural [25,26] and man-made composites, such as brick masonry). The identification of adequate cohesion laws between particles in direct contact is of fundamental importance to obtain an accurate modeling of the continuity of matter [27]. Some recent DEM approaches describe the cohesive bonds using a cohesive beam model—based on Euler/Bernoulli theory—that also includes coupling terms between bending and tangential effects [28]. In 3D domains, this results in a six-component vector of the generalized forces acting as attractive internal forces between two particles in contact, with one normal component to counteract the relative normal displacement in traction, two tangential components to counteract the relative tangential displacements, and three moment components to prevent both bending and twisting effects. The cohesive beam model also provides more realistic crack patterns [27,29,30] than previous spring models [31,32] and is suitable for modeling the heat-induced damage in composites [33,34].

This paper provides the basic principles and shows some numerical results of the DECM (Discrete Elements Cell Method), which is the first numerical method born from the coupling of the DEM with the CM, to obtain a multi-scale analysis of the continuum and, in particular, of periodic composite continua. The DECM code (written in MATLAB by the author) is completely parametric and allows the user to define the number, geometry, and arrangement of the discrete elements.

In order to provide the reader with a complete overview on the subject, Section 2 frames the DECM in the wider field of DEM models and highlights some similarities and differences between the new numerical method and previous DEM approaches. Section 3 deals with an early CM code, in which all the main features of a DECM approach for crack propagation problems in multi-material domains are already clearly recognizable, even if not explicitly framed in the context of a DEM approach. In that early numerical model, the interfaces between different materials define the geometry of the distinct elements and the code models the overall behavior as a contact problem between bodies made of different materials. The DECM approach proposed in Section 4 overcomes this way of considering discrete elements, since the new discrete elements are not necessarily homogeneous and can include one or more sub-domains of different materials. Therefore, the contact points do not necessarily separate domains of different materials. This new approach allows a multi-scale modeling of periodic composite continua.

The main advantage of a DECM approach over a CM approach is to reduce the computation time. In fact, as for FEM, the computation time in both the CM and the DECM is directly associated with the degrees of freedom of the model, which depend on the size of the discretization mesh. Therefore, the number of grid-points being equal, by dividing the domain into discrete elements and performing the static/dynamic analysis on the single discrete elements taken individually, the degrees of freedom and the dimensions of the stiffness matrices decreases with the number of discrete elements used, which takes less time to calculate. Moreover, if the structure of the numerical model allows the use of a number of processors to perform the static/dynamic analysis simultaneously on several discrete elements, the calculation time can decrease further, even drastically.

## 2. DEM, CM, and DECM Approaches to Model the Continuum

In DEM models for continuous media, a geometric form reproduces the shape of the continuum and the user or a specially generated code fills the geometrical form with particles. However, the difficulty in forming the geometry of the model is one of the major disadvantages of DEM analyses of continua. Furthermore, since large-scale problems require excessive computation times, even in the case of continuous media, the numerical simulations allowed by a DEM are generally small-scale. In addition, discrete modeling usually requires a time-consuming calibration phase to match the micro-scale and macro-scale parameters.

It is worth noting that, since the calibration depends on the specific geometry as well as on the macroscopic properties, it is necessary to perform it only once for each type of test. This allows the user to avoid performing the calibration every time by taking advantage of some numerical abacuses that provide the microscopic properties corresponding to the desired macroscopic behavior. However, the computation time related to the calibration process remains undoubtedly one of the major concerns in DEM analyses. Lastly, since the ideal particle size is similar or proportional to the actual grain size but it is neither practical nor possible to model each particle, the model needs a particle scaling process and this may require additional time-consuming calibrations.

Although originally developed for geomechanics, the DEM numerical codes also found applications in civil engineering especially for the modeling of masonry structures [35,36]. In fact, masonry lends itself well to modeling with the DEM because it is a particular type of periodic composite continuum, obtained from the regular arrangement of bricks (inclusions) and mortar (matrix).

There are at least two possibilities to model unreinforced masonry (URM) with a DEM numerical code. The first possibility is a micro-modeling approach and consists in modeling a masonry element by means of distinct blocks and joints, represented by face-to-face contacts through assigned contact points. The blocks can be rigid or, in more refined analyses, deformable [37,38,39]. In the second case, the DEM model solves the problem in large displacements for the joints and small deformations for the blocks. This second approach requires a preliminary assessment of the minimum number of contact points to obtain the correct solution. Furthermore, the finite elements used for the internal mesh of the deformable blocks are poorly performing [2]. Therefore, the DEM models with deformable blocks are not useful for studying the stress field within the blocks. This does not prevent the method from correctly capturing the collapse mechanisms due to sliding, rotation, and impact. However, being a micro-modeling approach, this method is appropriate for the detailed analyses of small masonry models and not of entire structures, apart from the cases of very simple structural geometries.

The second possibility starts from the experimental observation to obtain a meso-modeling and macro-modeling of masonry structures. Traditional URM buildings usually respond to seismic actions with the activation of recurring damage mechanisms, which depend on the building typology [40,41]. These damage mechanisms generate cracks that follow the interfaces between mortar and bricks and divide the URM structures into macro-elements. Whenever the masonry blocks that are most likely to form are clearly identifiable for the different loading conditions, a DEM approach is more suitable than an FEM approach to model the interaction between the macroscopic blocks and their separation [42,43]. The equations of the problem are still the differential equations of motion, integrated in the time domain even for static and quasi-static loads. The DEM codes belonging to the second strategy consider the macroscopic masonry blocks as rigid bodies in equilibrium with each other, which interact through unilateral elasto-plastic contact elements that follow a Coulomb slip criterion to simulate contact forces. However, although ignoring the deformability of the blocks may be appropriate for granular materials [44], things can be different for non-granular materials, where the deformability of large blocks can modify the collapse mechanism. In the specific case of real masonry buildings, a numerical analysis performed on macroscopic rigid blocks is not appropriate whenever the building has a box-type behavior [45,46,47], which allows the redistribution of stresses between structural elements of different stiffnesses and strengths. In particular, a DEM analysis with macroscopic rigid blocks could underestimate the strength capabilities of a real building by predicting collapse conditions not confirmed by the experimental results [48].

The misevaluation of the stress not only characterizes DEM analyses with macroscopic rigid blocks, but also the 3D modeling of the continuous medium with a dense granular packing and a cohesive beam model. In the latter case, the local stress field is highly heterogeneous even in the case of a theoretically homogeneous field. This leads to an overestimation of the local stress, which triggers an early crack initiation and accelerates crack propagation. A successful technique to reduce the heterogeneity of the stress field is to evaluate the stress of each discrete element (DE) by taking into account the contributions of the DEs contained in an appropriate meso-scale neighborhood—called the Halo [49]—of the DE. However, since this technique requires a number of DEs per Halo of the order of $10^{3}$ to achieve convergence in the stress values, the Halo approach is not suitable for use with macroscopic rigid blocks.

**Figure 1:**
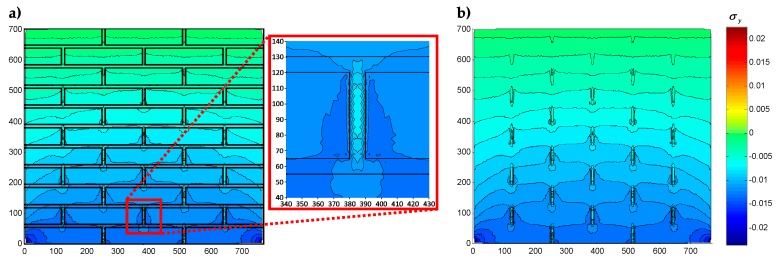
Isolines of the normal stress $\sigma_{y}$ (the y-axis runs vertically): (**a**) With and (**b**) without the arrangement of the bricks in the foreground (stress values in $N/{\mathrm{mm}}^{2}$ and linear measurements in mm).

A further micro-modeling approach that has proved to be suitable for modeling matrix/inclusions interactions is the CM [5,6,7,8,50,51,52,53]. As far as the modeling of URM structures is concerned, the CM treats the masonry as a bi-material consisting of mortar and bricks and provides descriptions up to the scale of the individual bricks [52]. This allows an accurate investigation of the interaction between mortar and bricks, which shows that the vertical stress of bricks subjected to the masonry’s own weight is greater near the vertical mortar joints and decreases when passing from the bricks to the mortar of the adjacent vertical joint (Figure 1a). In other words, the mortar of the vertical joints exerts an effect on the bricks on the left side and on the right side, which is similar to the negative skin friction (NSF), exerted by soft soils on concrete piles [54,55]. Unlike what happens in soft soils, the cause of the negative skin friction on the bricks is not the consolidation over time, but the different stiffness of the two materials (mortar and brick) that undergo the same vertical displacements when subjected to a vertical load.

As already highlighted in Section 1, the ability of the CM to model domains made up of different materials without requiring any homogenization technique derives from the direct algebraic formulation of the CM. The purely algebraic approach of the CM is a direct algebraic approach in the sense that it is not induced by the differential formulation, which happens for the discrete formulations. In fact, the latter make use of one of the various discretization methods to derive the finite formulation from the differential formulation. In particular, the CM shares some features with the lattice models—such as the Delaunay/Voronoi dual tessellation used in some lattice models for two-dimensional problems [56,57,58]—but, unlike them, the CM provides a direct finite formulation of field equations without taking the differential formulation as a starting point [59].

The micro-scale analysis allowed by the CM makes the CM useful for providing detailed descriptions of discrete elements assembled in complex geometries, such as for the Universal Distinct Element Code (UDEC) [60]. This gives rise to the new numerical approach presented in this paper for the first time, namely, the DECM. Compared to a DEM analysis, the DECM has the advantage of taking into account the deformability of the discrete elements without incurring problems of poor performance of the internal mesh since the mesh used by the CM allows the description of the stress field inside the blocks.

## 3. Basic Principles of the Discrete Elements Cell Method (DECM)

According to the original definition proposed by Cundall and Hart [61], a discrete element method is any numerical technique that allows finite displacements and rotations of discrete bodies—including complete detachment—and recognizes new contacts automatically, as the simulation progresses. In this definition, Cundall and Hart made no reference to the type of solving equations. Therefore, the CM simulations of the pullout test (specifically, of the Lok-Test [62]) and thermal debonding of tiles in radiant heat floors [63] are early examples of Discrete Element modeling with the CM, even if they do not use the explicit, time domain solution of the original equations of motion (not the transformed, modal equations), which is typical of the DEM models. In fact, unlike any other DEM approach, the static solution of the DECM is a direct achievement and not the result of a dynamic relaxation technique, which consists of introducing viscous damping to obtain steady state solutions of a dynamic problem [64,65]. Incidentally, it is worth noting that the explicit time stepping DEM algorithms are very effective only for quasi-static analyses, whereas, in dynamic analyses, the time steps are often very small due to numerical stability requirements (impossibility to apply mass scaling) [66]. Actually, the maximum stable time step depends on the minimum Eigen period of the total system. However, since it is not practical to perform Eigen value analyses in DEM simulations, determining the critical time requires some approximations [11].

**Figure 2:**
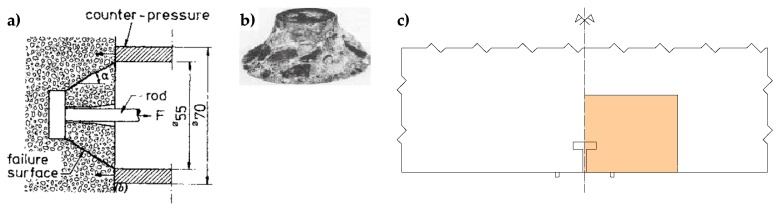
The Lok-test: (**a**) Geometric characteristics of the insert and counter-pressure ring for the pullout test. (**b**) Shape of the extracted portion in concrete solids. (**c**) Shape of the modeled domain.

**Figure 3:**
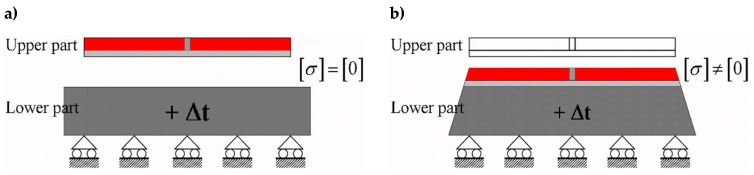
CM modeling of temperature variation in the sub-base of a radiant heat floor: Shape of the sub-domains (**a**) before and (**b**) after restoring compatibility between the sub-domains.

In the simulations of both the pullout test [62] and the detachments in radiant heat floors [63], the DECM provides a static numerical solution in the space domain by enforcing both equilibrium and compatibility between the sub-domains, which are equivalent to the DEM blocks. In this early formulation of the DECM, the boundaries of the sub-domains are the interfaces of discontinuity of the constitutive properties (Figure 2c and Figure 3).

Therefore, a DECM analysis addresses the static problem between sub-domains of different materials as a static contact problem [67], which is easy to manage in the CM and not trivial in the differential formulation [68,69,70,71,72,73,74,75].

As for the DEM and contrary to the Finite Element (FE) micro-models, the DECM does not require definition of joints or interface elements, but uses a point contact approach. Compared to the use of joints or interface elements, the point contact approach is more versatile and facilitates the analysis in the large displacement range [76]. In fact, it allows taking into account the variations in the connectivity of the system in large displacements by periodically updating the type, location, and orientation of contacts.

A typical drawback of DEM approaches is that the accuracy of the distribution of stresses depends on the number of contact points. For instance, a low number of contact points can give results that deviate from the correct solution. On the other hand, the computational cost of a dynamic relaxation technique for static problems can increase significantly with the number of contact points. This requires finding a compromise between reasonable computation time and required level accuracy [37].

**Figure 4:**
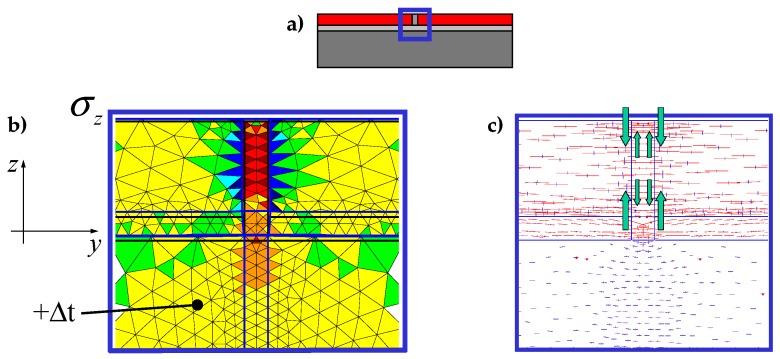
Modeling of radiant heat floors: (**a**) Detail of a modeled joint, (**b**) plot of the vertical stresses (positive stresses in warm colors and negative stresses in cool colors), and (**c**) principal directions of stress.

In the DECM, conversely, the use of a direct static solution does not impose serious limitations on the number of contact points. Therefore, in building the discretization mesh along the block interfaces, the numerical model generates pairs of opposite nodal points (twin nodes) in which each is a contact point. This allows the numerical model to provide the same solution obtained in the case of material continuity when no relative displacements occur along the interfaces. Consequently, at the interfaces between sub-domains in direct contact, the DECM offers the same degree of detail as a CM analysis (performed on a single domain consisting of several materials). In particular, similarly to what happens in the CM model [52], in the DECM model the condition of perfect adherence together with the difference in stiffness between the inclusions (the tiles) and the matrix (the grouting) force the tiles to lengthen the grouting along the vertical interfaces, while the grouting compresses the tiles along the vertical interfaces ([63], Figure 4b). Consequently, both the principal stresses in the grouting of the vertical joints are positive (tensile stresses), while the vertical principal stress in the tiles is negative (compression stress) (Figure 4c). Furthermore, the tile/grouting interaction at the corners of the tiles modifies the principal directions of stress in both the grouting and the subbase (Figure 4c), which provides a description of the corner effect [77,78].

The good agreement between the results provided by the CM on a single domain and by the DECM on a domain composed of several discrete elements is proof that the reaction forces of the twin nodes are suitable for adequately modeling the continuity of matter without having to introduce additional cohesion laws.

**Figure 5:**
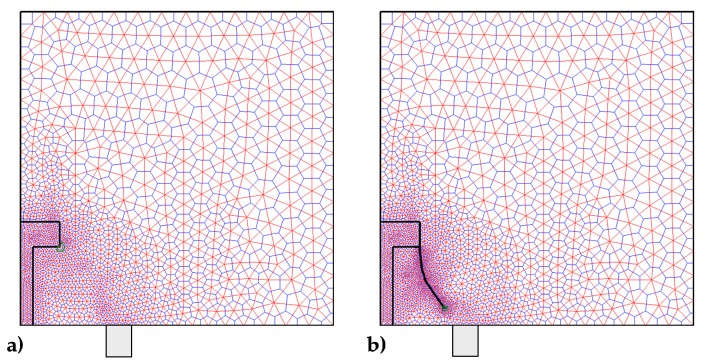
Shape of the domain modeled for the Lok-test simulation: (**a**) At the beginning of the computation and (**b**) after several steps of crack propagation.

**Figure 6:**
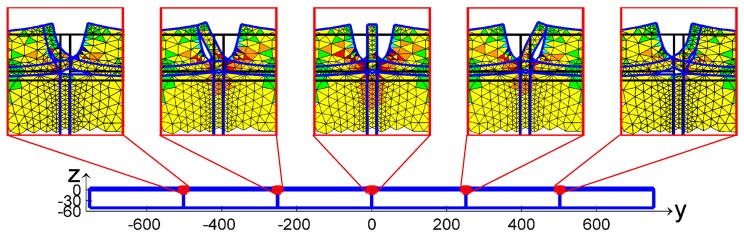
Details of the detachments on the joints of the tiles due to temperature variation.

The failure criterion discussed in Section 3.2 then allows the numerical model to activate crack propagation in the sub-domains (Figure 5) or nodal relaxation of the contact points on the interfaces between sub-domains (Figure 6). Since both crack propagation and nodal relaxation modify the geometry of the sub-domains and can generate new sub-domains, the DECM model updates the geometry and number of the blocks at each iteration. Consequently, unlike the DEM meso-approaches and macro-approaches for masonry structures, a DECM model does not require a preliminary knowledge of the shape and number of all the blocks generated by the damage and failure mechanisms. The detection of eventual contact points along the new surfaces generated by the propagation of the cracks takes place according to the same contact detection algorithm used for the blocks of the initial geometry (Section 3.1).

### 3.1. Contact Detection Algorithm

As in the DEM models and unlike what happens in the FEM models, the DECM sub-domains can lose existing contacts and create new ones. Actually, the contact detection algorithm is the most challenging and time-consuming part of the discrete element codes [79,80]. Even in DECM, the computation time depends sensitively on this algorithm.

**Figure 7:**
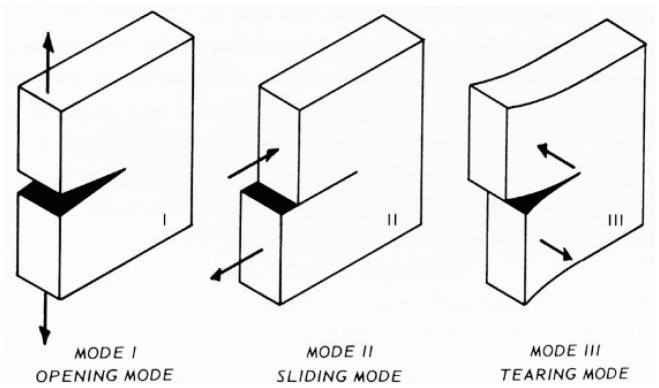
The three modes of propagation in fracture mechanics.

The contact detection algorithm of DECM is the same one used in the former CM codes, originally developed to model strain softening in concrete elements [59]. The need to use a contact detection algorithm in an interface problem arises whenever the displacements along the interface occur in Mixed Mode loading [81]. In plane problems, this means that the opposite edges of the interface move in Mode I (opening mode, Figure 7) for a portion of the interface while, for the remaining part, they move in Mode II (sliding mode, Figure 7). In the absence of a contact detection algorithm, the two opposite edges that are in Mode II would overlap, which leads to the unrealistic situation of matter compenetration. Actually, the absence of contact forces along the edges in Mode II also causes matter compenetration in part of the interface that is in Mode I and, precisely, in the part that is closer to the point of separation between Mode I and Mode II. Evaluating the contact forces along the edges in Mode II is not easy from the numerical point of view because the point of separation between the two modes of propagation is an unknown of the propagation step. Therefore, the contact detection algorithm must identify the point of separation for each calculation step, which differs from the previous one for the value of impressed displacement or impressed load (the numerical model can work in both displacement control and load control). The identification of the separation point occurs in an iterative way through a stabilization procedure. At the beginning of the stabilization for a given calculation step, the forces on the nodes of the interfaces are the same as in the previous calculation step (they are equal to zero for the first calculation step). Then, the contact detection algorithm calculates the relative displacements for all twin nodes: a positive value of relative displacement means that the twin nodes are not in contact and a negative value of relative displacement means that the two opposite edges of the interface are overlapping. Thus, a negative value of relative displacement is a measure of matter compenetration.

The number of twin nodes that compenetrate in this calculation phase provides the first estimate of the number of nodes in Mode II. The code modifies the displacements of these nodes to align them along the deformed interface. This involves the introduction of constraints along the direction orthogonal to the deformed interface, while a friction law regulates the displacements along the deformed interface. The constraints introduced along the interface together with the law of friction allow the opposite edges of the interface to interact, which establishes a contact constitutive law for the twin nodes in Mode II. The DECM contact constitutive law is the algebraic equivalent of the FEM contact elements used to describe the sliding contact with the differential formulation [82,83,84].

The phase of node alignment along the deformed interface is the most delicate of the entire procedure since the nodes that are actually in Mode II are only a subset of the nodes that compenetrate at the beginning of the stabilization procedure. Therefore, if the code simultaneously aligns all the overlapping nodes of the first estimate, the numerical solution may not converge.

**Figure 8:**
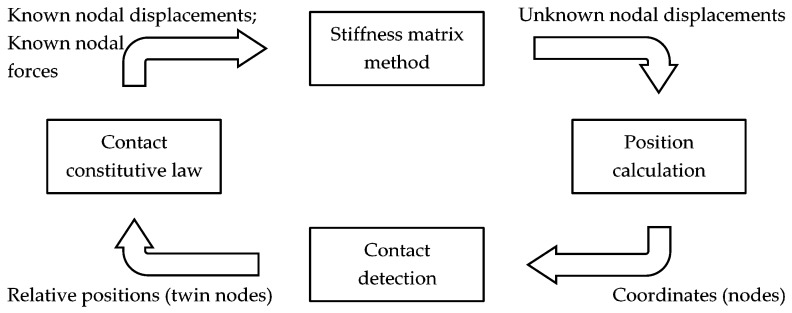
The DECM stabilization procedure to take into account both the compatibility and the equilibrium on the interfaces.

In order to avoid numerical instabilities, the code operates on a pair of twin nodes at a time, starting from the pair with the maximum overlap. To be precise, the code imposes the first attempt displacement components on a single node of the pair (introducing the appropriate constraints), calculates the reaction forces for the same node, and applies balancing forces on the second node of the pair. However, since the displacement imposed on the first node of the pair is only the first estimate of the actual displacement, the reaction forces of the first estimate are not equal to the actual contact forces. Therefore, if the balancing forces on the second node were equal and opposite to the reaction forces of the first estimate on the first node, the numerical solution may not converge again. Consequently, the code uses a bisection technique to estimate the balancing forces, calculates the new positions of the nodes, adjusts the position of the constraints along the interfaces, and modifies the balancing forces iteratively (Figure 8), until the difference between the reaction and the balancing forces is less than or equal to a prefixed value. The iterative updating of forces and displacements allows the code to provide a numerical solution that respects both the compatibility of displacements and the equilibrium of forces for each pair of nodes in Mode II, along the interfaces. At the end of the stabilization procedure, the contact forces equalize the reaction forces of the introduced constraints.

Enforcing both compatibility and balance could cause the DECM contact detection algorithm to look similar to an implicit FEM analysis. However, unlike an implicit FEM analysis, the DECM code does not enforce equilibrium after compatibility at a later stage (for example, through expensive Newton-Raphson iterations). Actually, the CM uses two meshes of discretization [4,59] (Section 4.2) and enforces compatibility and balance simultaneously, on the geometric elements of the two meshes. In particular, the first mesh is a simplicial cell complex and contains one node of the second cell complex. The CM associates the configuration variables (geometric and kinematic) with the elements of the first mesh and the source variables (static and dynamic) with the elements of the second mesh. Therefore, the CM enforces the conservation laws on the geometric elements of the second mesh in the same stage in which it computes the displacements for the nodes of the first cell complex.

Furthermore, it is worth noting that the DECM stabilization procedure, outlined in Figure 8, is very similar to the iterative DEM calculation scheme, called the “calculation circle.” Actually, the only other difference apart from the use of the stiffness matrix method that replaces the law of motion lies in the variable, which is the time instant for the DEM and the difference between reaction and balancing forces for the DECM.

**Figure 9:**
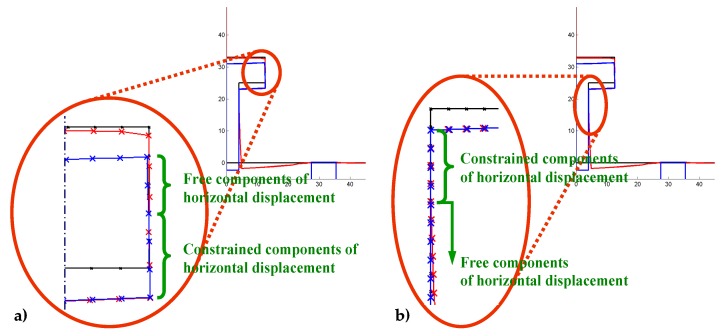
Deformed configuration and detail of the identified boundary conditions for the pullout test: (**a**) On the thickness of the disc and (**b**) on the rod.

After each alignment, the code re-calculates the relative displacements for the pairs of twin nodes, since the alignment of a single pair can reduce the total number of nodes in Mode II. At the end of the alignment procedure, there are no forces applied to the nodes in Mode I and the forces on the nodes in Mode II are equal to the contact forces. Figure 9 shows the nodes in Mode I and Modes II, identified on the two vertical interfaces of the pullout test by the contact detection algorithm.

The number of contact points changes at each step of the calculation, depending on how the boundary conditions modify.

In the case of crack propagation through a sub-domain (Figure 5), the contact detection algorithm also identifies the points of separation between Mode I and Mode II along the cracks. Even in this second case, the number of interactions depends on the boundary conditions.

### 3.2. Direction of Crack Propagation

The CM and DECM codes evaluate the condition for crack extension and the direction of crack propagation in the Mohr/Coulomb plane. The limit domain used for brittle materials is the domain of Leon, which is a parabolic approximation of the envelope of the limit Mohr’s circles constructed for any three-dimensional state of stress at a point. Compared to the linear Mohr’s envelope—consisting of the two common tangents to the two limit Mohr’s circles for uniaxial compression and uniaxial tension—the limit domain of Leon is better suited to the actual envelope of the limit Mohr’s circles in a triaxial traction. The equation of the limit domain of Leon in the Mohr/Coulomb plane is shown below.

(1) $$\tau_{n}^{2}=\frac{c}{f_{c}}\left(\frac{f_{tb}}{f_{c}}+\sigma_{n}\right),$$

where $\sigma_{n}$ and $\tau_{n}$ are, respectively, the normal and shear stresses on the attitude normal to the line n, c is the cohesion, $f_{c}$ is the compressive strength, and $f_{tb}$ is the tensile strength.

**Figure 10:**
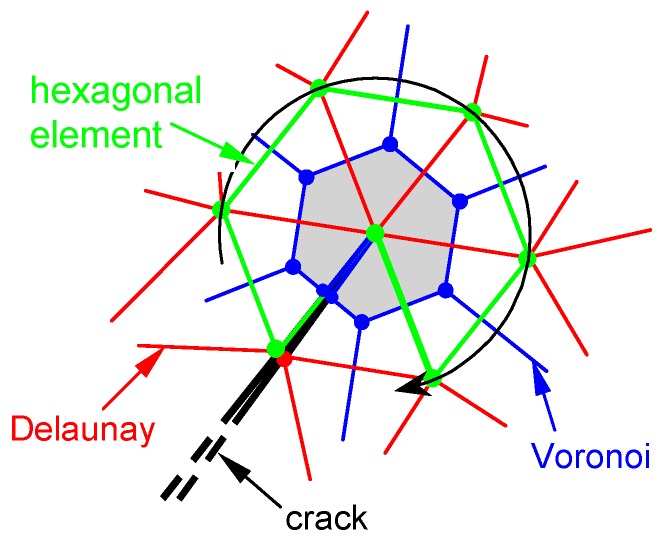
The regular Voronoi polygon generated on the crack tip.

Since the CM enforces the conservation laws on the Voronoi polygons—whose vertices are the circumcenters (Figure 10) or the barycenters of a Delaunay triangular mesh—in order to evaluate $\sigma_{n}$ and $\tau_{n}$ near the crack tip, the CM generates a regular Voronoi polygon on the crack tip. By computing the stresses for the nodes of the Voronoi mesh, the CM identifies the largest Mohr’s circle in the finite neighborhood of the crack tip.

**Figure 11:**
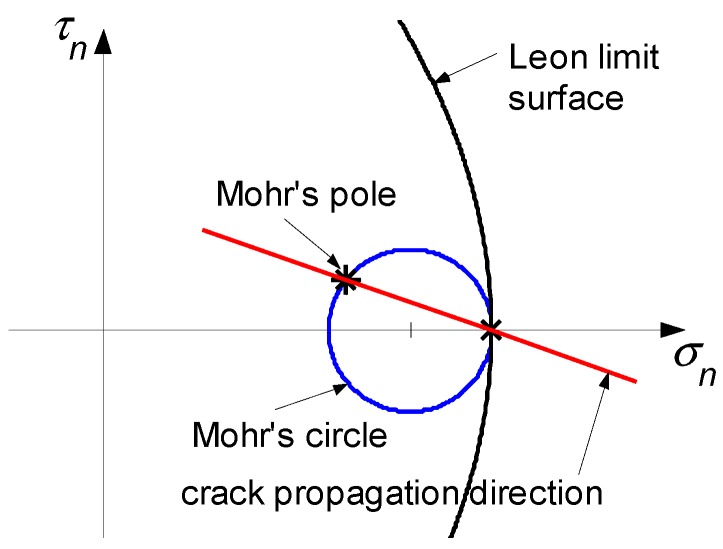
Limit domain of Leon in the Mohr/Coulomb plane.

For low external loads, the Mohr’s circles lie within the limit domain of Leon, with the radii of the circles increasing with growing loads. When the largest Mohr’s circle for the finite neighborhood of the crack tip becomes tangent to the limit domain of Leon, the crack extends. If the Mohr’s circle is completely contained in the positive half-plane of $\sigma_{n}$, as in Figure 11, there is only one point of tangency between the largest circle and the limit surface: the vertex of Leon’s parabola [85]. Otherwise, the points of tangency are two [62].

In the first case, the direction of crack propagation is the direction of the line joining the Mohr’s pole (also called the origin of planes) to the point where the Mohr’s circle is tangent to the limit domain (Figure 11). In the second case, the possible propagation directions are two, which actually activates depending on the constraint conditions along the directions.

**Figure 12:**
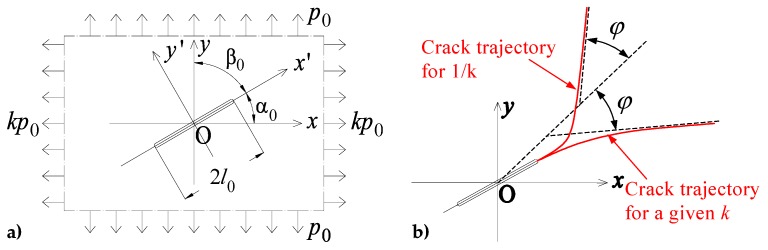
Concrete plate in biaxial tensile load: (**a**) Geometric characteristics and (**b**) crack trajectories for a given k and 1/k.

The concrete plate of Figure 12a—with two tensile loads $p_{x}=kp_{0}$ (parallel to the x-axis) and $p_{y}=p_{0}$ (parallel to the y-axis) applied at infinity and an initial straight crack of length $2l_{0}$ inclined by an angle $\alpha_{0}$ with respect to the x-axis—belongs to the first case. The crack propagation mode of a cracked plate subjected to tensile loading at infinity is brittle with the Mohr’s pole moving along the Mohr’s circle [86]. This modifies the propagation direction at each propagation step.

The parametric analysis for variable values of k and $\alpha_{0}$ shows that the crack trajectory tends to approach an asymptote inclined by an angle γ (with respect to the x-axis) that depends on k while it does not depend on the inclination $\alpha_{0}$ of the initial straight crack [87]. Moreover, the asymptotes of the crack trajectory for a given k and its reciprocal value, 1/k, are symmetric with respect to the bisector of the first quadrant in Figure 12a (Figure 12b).

(2) $$\gamma \left(\frac{1}{k}\right)=\frac{\pi }{2}-\gamma \left(k\right).$$

### 3.3. Constitutive Assumptions

The material parameters required for the DEM analysis of a continuum are the micro-mechanical parameters that define the behavior of a single particle, which are not directly related to the mechanical behavior of the continuum itself. In fact, by modeling the continuum as an assembly of discrete particles that interact through a contact constitutive law, the DEM approach separates the micro-scale from the macro-scale. From the numerical point of view, this is one of the typical disadvantages of using a DEM approach to model the behavior of continua, as it requires a preliminary numerical calibration to match the micro-scale and macro-scale parameters [88]. On the other hand, however, the assembly of discrete particles allows us to take into account the discontinuous nature of matter on the micro-scale.

Furthermore, what the experimenters usually neglect when performing material characterization tests is the damage suffered by a specimen during the test. Damage develops throughout the duration of a material characterization test instead of at the end of the test. Consequently, the specimen is a discontinuous medium and behaves on a macro-scale according to laws that are not constitutive in the strict sense, since they depend on the crack pattern generated by the damage. This means that identifying the constitutive properties of a material as the normalized macroscopic properties of a specimen made of that material is purely utopian [89].

Unfortunately, the technical standards on how to perform material characterization tests have not yet implemented this level of accuracy in the processing of experimental data. Consequently, the constitutive properties of the materials continue to be confused with the average properties. This is the main cause for which the FEM models for cohesive materials use complex constitutive relationships with a number of required parameters that increases with the complexity of the model. These parameters require calibration on the individual test and, at times, do not have clear physical meanings [90]. Furthermore, some specific problems are impossible to deal with the FEM approach even after an accurate calibration of the parameters. For example, it is impossible to model the softening effect with the FEM without running into problems of numerical instability. Conversely, the intrinsic separation between micro-scale and macro-scale of the DEM allows the identification of the elastic micro-properties that capture the softening effect on the macro-scale in both uniaxial compression and uniaxial tension [18].

Starting from a different viewpoint, even the DECM introduces a separation from the micro-scale and the macro-scale. Actually, unlike the DEM and similarly to the FEM, it is possible to introduce the laws of the continuous mechanical behavior directly into the DECM formulation. Therefore, with the DECM, it is no longer necessary to identify the microscopic interaction laws from the mechanical behavior of the continuum. However, the DECM does not use the same constitutive laws as the FEM because it takes into account the discontinuities induced by the damage.

Being unable to perform the DEM calibration on the micro-mechanical parameters, the DECM uses the results provided by the identification procedure of the effective law [91]. For brittle heterogeneous materials, this procedure identifies monotone strictly nondecreasing material laws, called “effective laws,” which makes use of a micro-seismic damage parameter, D, which allows the quantification of damage during the uniaxial compression tests [92]. Specifically speaking, using two probes for micro-seismic analyses glued on two diametrically opposite points of the middle cross-section of a cylindrical specimen and acquiring the velocity of the micro-seismic signal for the whole duration of a uniaxial compression test, the variation of the micro-seismic velocity provides a measure of the damaged parameter for each instant of time [93].

(3) $$D=1-\frac{V}{V_{0}},$$

where $V_{0}$ is the micro-seismic velocity at the beginning of the test and V is the micro-seismic velocity in the generic instant of time. Since V decreases during the test, D varies from 0 (no damage at the beginning of the test) to 1 (specimen crushing at the end of the test).

The procedure of the effective law uses the damage parameter provided in Equation (3) to identify the law of variation of the resistant area, $A_{res}$, defined as the portion of the middle cross-section that bears the external load.

(4) $$A_{res}=A_{n}\left(1-D\right),$$

where $A_{n}$ is the nominal area of the specimen.

The resistant area is smaller than the nominal area due to the propagation of macro-cracks, which gradually reduce the load-bearing capacity of the specimen throughout the uniaxial compression test.

The effective stress, $\sigma_{eff}$, is the ratio of the external load, N, to the resistant area, $A_{res}$.

(5) $$\sigma_{eff}=\frac{N}{A_{res}}=\bar{\sigma }\frac{A_{n}}{A_{res}},$$

where $\bar{\sigma }$ is the average stress.

(6) $$\bar{\sigma }=\frac{N}{A_{n}}.$$

**Figure 13:**
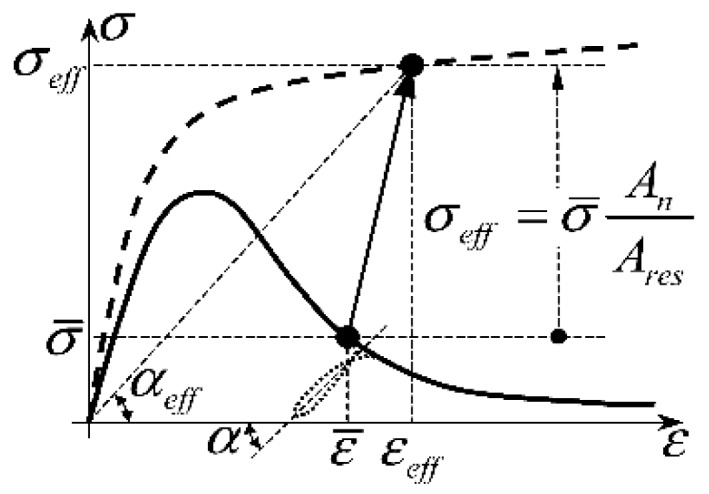
Identification of the effective properties starting from the average properties.

Given the effective stress, Figure 13 shows how to identify the effective strain, $\varepsilon_{eff}$ [91].

In conclusion, the procedure of the effective law identifies the micro-mechanical properties directly from the experimental data and not through a numerical calibration (which happens in the DEM). The main merit of this procedure is that the effective laws are size-effect and shape-effect insensitive [91], which means that there are no material parameters to calibrate on the individual test. Furthermore, unlike the traditional procedures to identify the constitutive laws, the identification procedure of the effective law does not introduce a mere scale factor between the load/displacement and the stress/strain curves, which, therefore, do not have the same shape. In particular, even if the load/displacement curves—and, consequently, the average stress/average strain curves—are softening, the effective stress/effective strain curves are not softening (Figure 13).

The CM modeling of the uniaxial compression test showed that the monotone effective laws together with the crack propagation criterion discussed in Section 3.2 are able to model the softening effect in the load/displacement curves of concrete specimens [94]. Furthermore, although the effective law is size-effect and shape-effect insensitive, the crack propagation modeling with the CM provides load/displacement curves in good agreement with the experimental results on cylindrical specimens of different slenderness in uniaxial compression [95] as well as on specimens of different shapes [96].

The long-range interaction [18] and the Halo approach [49]—both used to model the macroscopic behavior of composite continua—characterizes the DEM as a nonlocal model [97,98,99,100,101,102,103] in a broad sense. In fact, in nonlocal continua, the stress at a certain point is a function of the strain distribution over a certain representative volume of the material centered at that point and not a function of the strain at the same point [104]. This is equivalent to abandoning the principle of the local action of classical continuum mechanics [105]. The algebraic formulation of the CM also provides a simplification of the constitutive assumptions necessary to take into account nonlocality. Actually, the main consequence of avoiding the limit process at every stage of the algebraic formulation is that the length scales are associated with the CM variables, which are global variables. This provides the CM with an intrinsic nonlocal nature [53,106]. Therefore, the CM does not require any nonlocal constitutive law—not even a nonlocal contact constitutive law—to model nonlocality in continua. The numerical modeling with the CM is nonlocal in any case.

From the mathematical point of view, the algebraic formulation is similar to performing non-standard calculus, which is the modern application of infinitesimals—in the sense of non-standard analysis—to differential and integral calculus [50]. Non-standard analysis extends the real line, ℝ, to the hyperreal line, *ℝ, where each real number has a collection of numbers (hyperreals) infinitely close to it. The name given to a collection of hyperreals is “monad” or “halo” (not to be confused with the Halo of the Halo approach [49]). The standard part function associates to a finite hyperreal, x, the unique standard real number, $x_{0}$, which is infinitely close to it. Therefore, by performing the standard part function, we lose information about the halo in the same way we lose information about the curvature when we calculate a derivative. Thus, the extension to the hyperreal line provides the real numbers with an additional structure of infinitesimal lengths, which is then lost with the standard part function. Following the mathematical analogy, we can conclude that both the long-range interaction and the Halo approach are attempts to enrich the DEM formulations with length scales, in the same way that the nonlocal approaches enrich the FEM formulations. The enrichment with the length scales is actually mandatory to model continua correctly in the context of a differential formulation due to the higher order information lost in performing the limit process of the derivatives. The algebraic approach, on the contrary, does not need any enrichment because it is to the differential formulation as non-standard calculus is to calculus.

## 4. DECM for Periodic Composite Continua

From the numerical point of view, the code for periodic composite continua is a generalization of the numerical models used for the pullout test and the thermal debonding of tiles in radiant heat floors. In fact, even in the latter case, the domain is composed of several sub-domains that interact through the contact points on the interfaces. However, unlike previous DECM models, the crack propagation does not necessarily initiate at the interface between domains made with different materials. This changes the philosophy behind the DEM approach, since the use of discrete elements is now justified by the need to compensate for the extremely large number of degrees of freedom when the structure increases in size and complexity in order to make the CM micromodel useful for the global analysis of entire buildings. To this end, the structure of the new DECM model has parallel processing capabilities to reduce computation times further.

In order to show how the DECM uses the discrete elements to model periodic composites in two-dimensional problems, Section 4.1 deals with the simplest case of the absence of crack propagation. This choice derives from the need to illustrate in detail how the new numerical approach works and does not represent a limitation for the DECM. In fact, since the DECM is an improvement of the CM, the new numerical approach shares the tools for crack propagation with the codes already developed for the CM. These codes are able to self-identify the position of crack initiation, manage the propagation of several cracks that elongate simultaneously, and estimate whether or not one or more cracks bifurcate [52].

### 4.1. Two-Dimensional Problems

Due to the periodic nature of the composite continuum, it is possible to treat each unit of the periodic pattern as a single discrete element. Based on this idea, the DECM idealizes the two-dimensional continuum as a two-dimensional array of rectangular discrete elements, arranged in rows and columns. Along the common sides of adjacent discrete elements, the DECM code generates a series of twin nodes, which are pairs of nodes with the same coordinates (one node of the pair is on a discrete element and the other node of the pair is on the adjacent discrete element). On the twin nodes, the adjacent discrete elements share the same boundary conditions in terms of both displacements and loading conditions. As explained in Section 3, this enforces material continuity between the discrete elements and generates the same numerical solution obtained for the non-discretized two-dimensional domain. Since the twin nodes can also relax, they behave like the contact points of the DEM models, with the advantage that the reaction forces of the twin nodes make the use of cohesive models at the interfaces between discrete elements no longer necessary to model the continuity of matter. Therefore, the reaction forces of the DECM twin nodes play the same role as the generalized forces in the cohesive beam model of the DEM approaches [28].

The DECM code examines the discrete elements iteratively, forms and stores the stiffness matrices of the discrete elements in separate workspaces, and performs the static analyses in separate workspaces. Therefore, unlike the FEM, the DECM code does not assemble the stiffness matrices of the single elements to construct a global stiffness matrix for the entire domain, but works on the single workspaces iteratively, updating the boundary conditions on the common sides up to numerical stabilization. Despite the need for numerical stabilization on the common sides, the use of small stiffness matrices leads to a significant reduction in computation time compared to a CM analysis on the whole domain. Moreover, different processors can work on different workspaces in parallel, which further reduces computation time.

In order to enforce both equilibrium and compatibility on the common sides, the DECM code operates on the single discrete elements performing stabilization cycles on both the rows and the columns. In particular, the DECM code processes the elements of each row twice. The first time, the code processes the elements of the row from left to right (that is, with an increasing value of the column index), while, the second time, it processes the same elements from right to left (with a decreasing value of the column index). In the iteration with an increasing value of the column index, the code imposes the forces on the left sides of the elements and calculates the forces on the right sides (where the displacements are known). In the iteration with a decreasing value of the column index, the code imposes the displacements on the right sides and calculates the displacements on the left sides (where the forces are known). A stabilization cycle then enforces both equilibrium and compatibility between the $n_{c}$ elements of the same row. Specifically speaking, in each stabilization iteration, the code uses a bisection technique for the imposition of both the forces and the displacements on the $n_{c}$ elements.

- The forces imposed on the nodes of the left side of the (j+1)-th element, with $1\leq j<n_{c}$, are half the differences (semi-differences) between the forces already present on the nodes and the forces calculated for the twin nodes on the right side of the j-th element,
- The displacements imposed on the nodes of the right side of the (j−1)-th element, with $1<j\leq n_{c}$, are half the sums (semi-sums) of the displacements already calculated for the nodes and the displacements calculated for the twin nodes on the left side of the j-th element.

The bisection technique for the forces uses a semi-difference instead of a semi-sum because the forces calculated for the nodes on the right side of the j-th element are reaction forces, which the code applies with the opposite sign on the left side of the (j+1)-th element to comply with the action–reaction principle.

**Figure 14:**
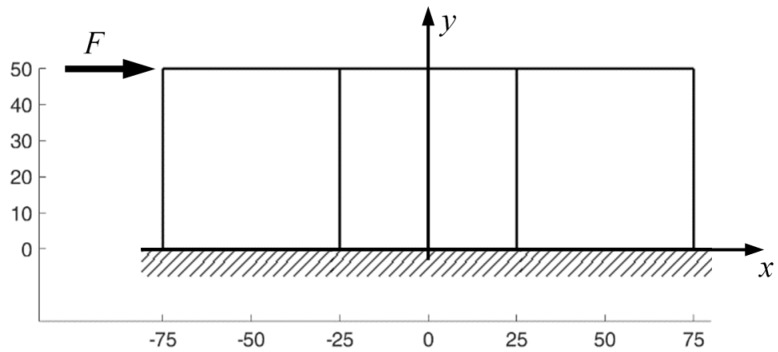
Geometry of a two-dimensional domain consisting of one row of three square elements (linear measurements in mm).

The two-dimensional domain in Figure 14 consists of a single row of three square elements with displacements constrained in both directions on the nodes of the lower sides. Although the DECM uses the non-linear elastic relationships identified by the procedure of the effective law (Section 3.3) to show how the DECM handles the discrete elements in the left-to-right-to-left procedure we will now consider the simplest case of linear elasticity. The Young’s modulus and Poisson’s ratio of the material are $E=2\cdot 10^{7}N/m^{2}$ and ν=0.3, respectively. Furthermore, the loading condition consists of a point load to emphasize the ability of the CM to handle concentrated forces easily, unlike the differential formulation. In particular, the point load is a horizontal force F=500 N, applied to the upper left node of the first element from the left in Figure 14.

**Figure 15:**
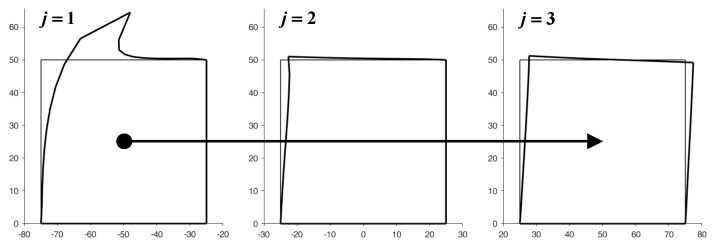
First iteration from left to right: the j-th element generates the forces on the left side of the (j+1)-th element, with j=1,2 (Thin line: undeformed configurations. Thick line: deformed configurations. Amplification factor of the displacements: k=200 ).

Figure 15 illustrates the deformed configurations of the three elements of Figure 14 at the end of the first iteration from left to right. In this first iteration, the displacements of the nodes on the right sides are equal to zero for all the elements apart from the last of the row (the third from the left in Figure 15). By means of the bisection technique described above, the reaction forces that nullify the displacements on the right side of the j-th element, with j=1,2, provide the first estimate of the forces applied to the left side of the (j+1)-th element.

In the iteration from the right to the left side, the code uses the bisection technique to improve the estimate of the displacements for the nodes on the right side of the (j−1)-th element with j=3,2. The new estimate of the displacements modifies the boundary conditions for the nodes of the right sides in the subsequent stabilization iteration. At the end of the stabilization cycle on the discrete elements, the left-to-right-to-right iterations guarantee both equilibrium and compatibility on the inner vertical sides. This restores the continuity between the discrete elements of the same row.

**Figure 16:**
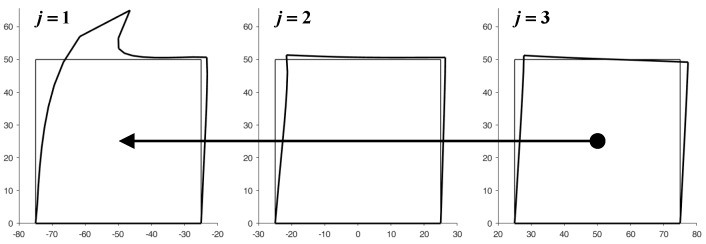
First iteration from right to left: the j-th element generates the displacements on the right side of the (j−1)-th element, with j=3,2 (Thin line: undeformed configurations. Thick line: deformed configurations. Amplification factor of the displacements: k=200 ).

**Figure 17:**
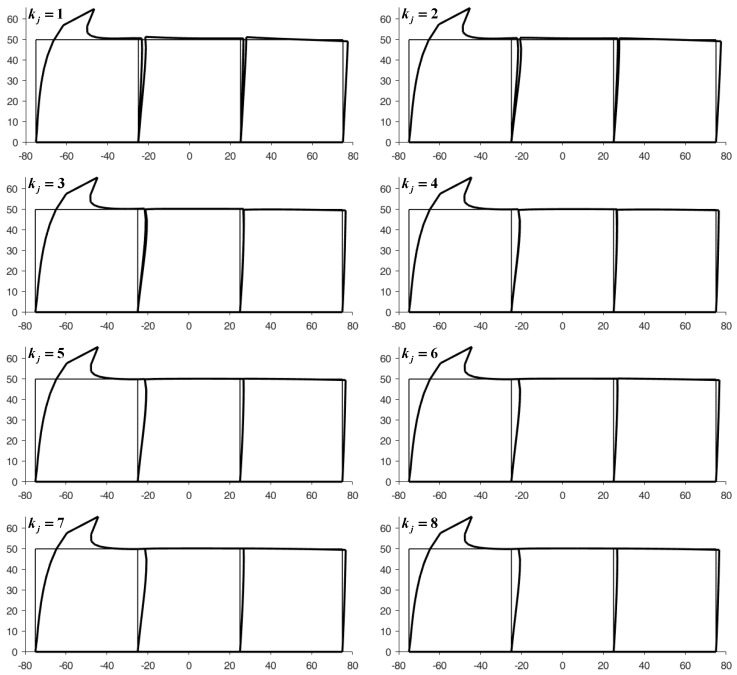
Stabilization cycle on the elements of the same row: deformed configurations provided by the $k_{j}\text{-}\mathrm{th}$ left-to-right-to-left iteration, for $1\leq k_{j}\leq 8$ (Thin line: undeformed configurations. Thick line: deformed configurations. Amplification factor of the displacements: k=200 ).

Figure 16 shows the displacements of the discrete elements at the end of the first iteration from the right to the left side, while Figure 17 provides the deformed configurations of the discrete elements for the first eight left-to-right-to-left iterations.

**Figure 18:**
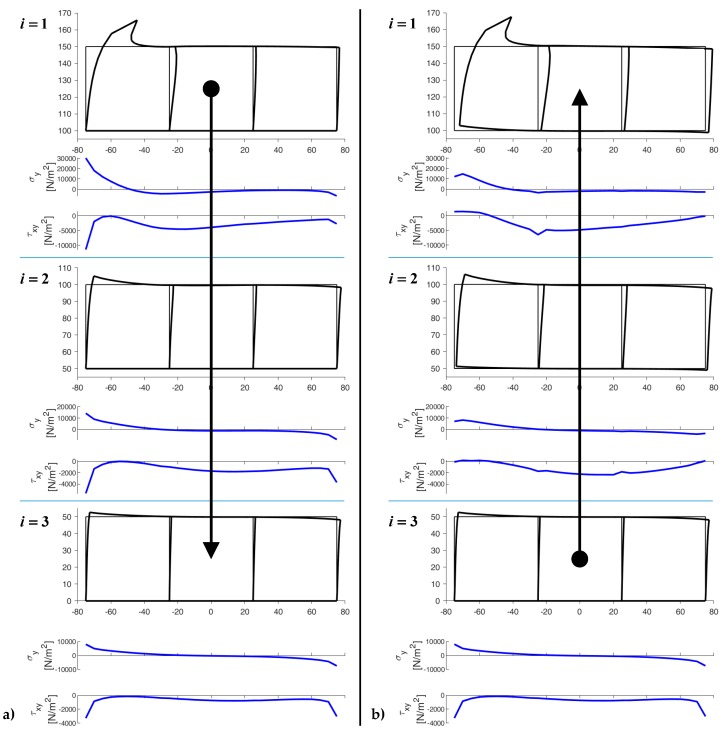
Deformed configurations and stress analysis on the constrained sides (the y-axis runs vertically) for the first top-to-bottom-to-top stabilization cycle on the rows. (**a**) In the first iteration from top to bottom, the i-th row generates the forces on the upper sides of the (i+1)-th row, with i=1,2. (**b**) In the first iteration from bottom to top, the i-th row generates the displacements on the lower sides of the (i−1)-th row, with i=3,2 (Thin line: undeformed configurations. Thick line: deformed configurations. Amplification factor of the displacements: k=200 ).

When the two-dimensional domain consists of more than one row, the DECM code performs the left-to-right-to-left stabilization on the rows twice (Figure 18). The first time from top to bottom (with an increasing value of the row index) and the second time from bottom to top (with a decreasing value of the row index).

In the iteration with an increasing value of the row index, the code imposes the forces on the $n_{c}$ upper sides of the rows and calculates the forces on the $n_{c}$ lower sides (where the displacements are known). In the iteration with a decreasing value of the row index, the code imposes the displacements on the $n_{c}$ lower sides and calculates the displacements on the $n_{c}$ upper sides (where the forces are known). A further stabilization cycle then enforces both equilibrium and compatibility between the $n_{r}$ rows, using a bisection technique to estimate both the forces and the displacements.

- The forces imposed on the nodes of the $n_{c}$ upper sides of the (i+1)-th row, with $1\leq i<n_{r}$, are half the differences (semi-differences) between the forces already present on the nodes and the forces calculated for the twin nodes on the $n_{c}$ lower sides of the i-th row,
- The displacements imposed on the nodes of the $n_{c}$ lower sides of the (i−1)-th row, with $1<i\leq n_{r}$, are half the sums (semi-sums) of the displacements already calculated for the nodes and the displacements calculated for the twin nodes on the $n_{c}$ upper sides of the i-th row.

Even in this second case, the bisection technique for the forces uses a semi-difference instead of a semi-sum to comply with the action–reaction principle.

For a two-dimensional domain made of three rows and three columns with displacements constrained in both directions for the lower nodes of the third row and a horizontal force F=500 N applied to the upper left node of the first row ($E=2\cdot 10^{7}N/m^{2}$ and ν=0.3), the first iteration from top to bottom provides the deformed configurations in Figure 18a. In this first iteration, the displacements of the nodes on the lower sides are equal to zero for all the rows.

**Figure 19:**
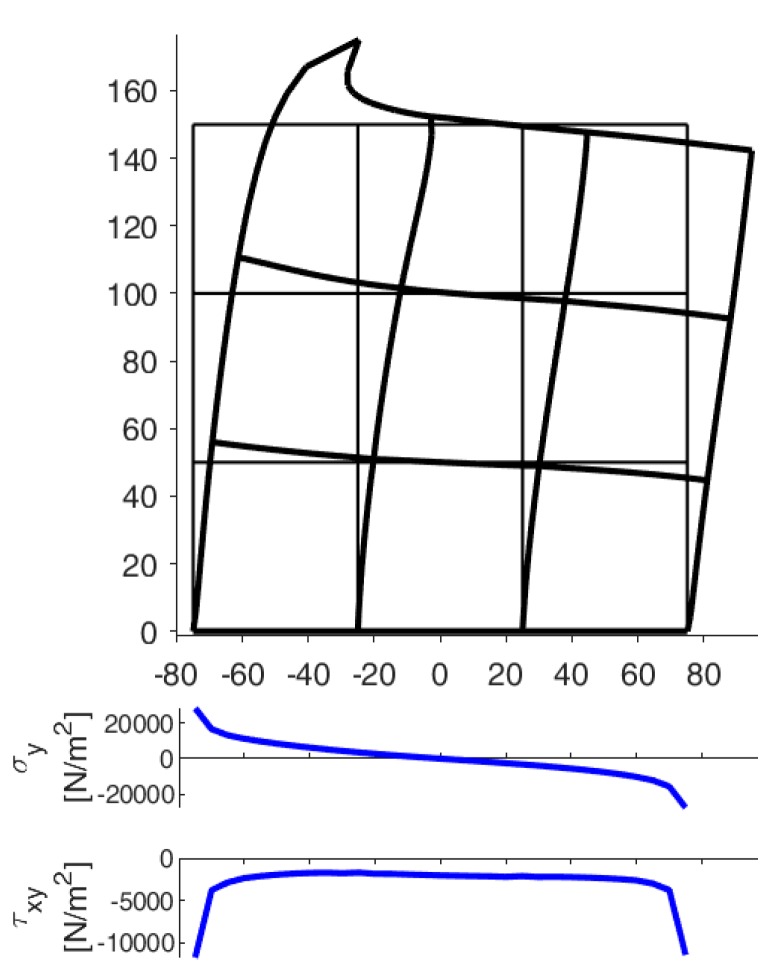
Deformed configurations of the nine discrete elements and stress analysis on the lower constrained sides (the y-axis runs vertically), after 15 top-to-bottom-to-top stabilization cycles on the rows (Thin line: undeformed configurations. Thick line: deformed configurations. Amplification factor of the displacements: k=200 ).

By means of the bisection technique for the rows, the reaction forces that nullify the displacements on the $n_{c}$ lower sides of the i-th row, with i=1,2, provide the first estimate of the forces applied to the $n_{c}$ upper sides of the (i+1)-th row. In the first (Figure 18b) and subsequent iterations from bottom to top, the code then uses the bisection technique for the rows to improve the estimate of the displacements for the nodes on the $n_{c}$ lower sides of the (i−1)-th row, with i=3,2. The new estimate of the displacements modifies the boundary conditions for the nodes of the lower sides, which is useful for the subsequent stabilization iteration. At the end of the stabilization cycle on the rows, the top-to-bottom-to-top iterations guarantee both equilibrium and compatibility on the inner horizontal sides. This restores the continuity between the rows (Figure 19).

The values of normal stress, $\sigma_{y}$, and shear stress, $\tau_{xy}$, on the lower sides in Figure 19 are greater (in absolute value) than those of the third row in Figure 18 because the stresses propagate progressively from the upper application point to the lower constraint as the stabilization procedure proceeds. The final distribution of $\sigma_{y}$ on the lower constraint is skew-symmetric—as it must be for equilibrium reasons, to comply with the loading condition (no external load along the y-axis)—while the $\tau_{xy}$ are symmetric.

The DECM code also provides the values of the three stress components within the discrete elements. Section 4.2 shows the comparisons between the values of $\sigma_{x}$, $\sigma_{y}$ and $\tau_{xy}$ obtained for the deformed configurations in Figure 19 and the stress values given by the DECM for an array with the same geometric characteristics and one round inclusion within each discrete element.

The stabilization cycles for both the elements (of the same row) and the rows are “while” loops, which end when the maximum relative displacement between twin nodes becomes less than a predetermined value. Since the displacement estimate for a given stabilization iteration is better than for the previous iteration, the number of (internal) iterations needed for the stabilization of the elements (of the same row) decreases at each (external) iteration on the rows. This increases the stabilization speed at each external iteration.

For large two-dimensional domains, it is possible to arrange the discrete elements in two-dimensional arrays of two-dimensional arrays (the internal arrays) and use different processors to perform the computation on different (internal) arrays simultaneously. By using a bisection technique for the twin nodes of adjacent (internal) arrays, this allows parallel computing with the DECM.

### 4.2. The Effect of the Inclusions for Shear Loads

Each discrete element can be non-homogeneous or made up of different materials. In the case of a two-dimensional domain consisting of a matrix with uniformly distributed inclusions, the discrete element that generates the entire domain is a rectangular element with one inclusion in the center.

**Figure 20:**
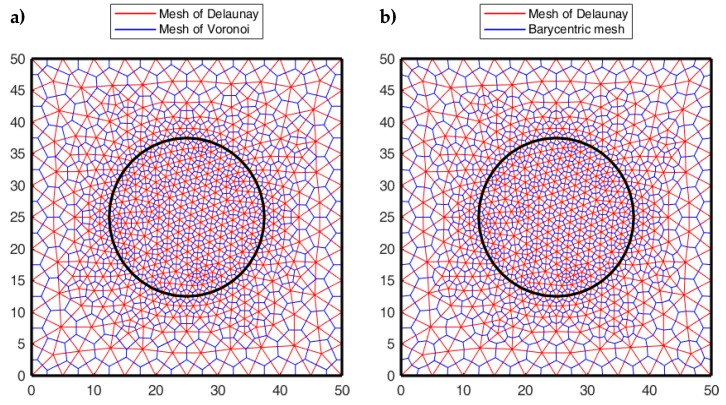
Generation of the second mesh, using (**a**) the circumcenters and (**b**) the barycenters.

**Figure 21:**
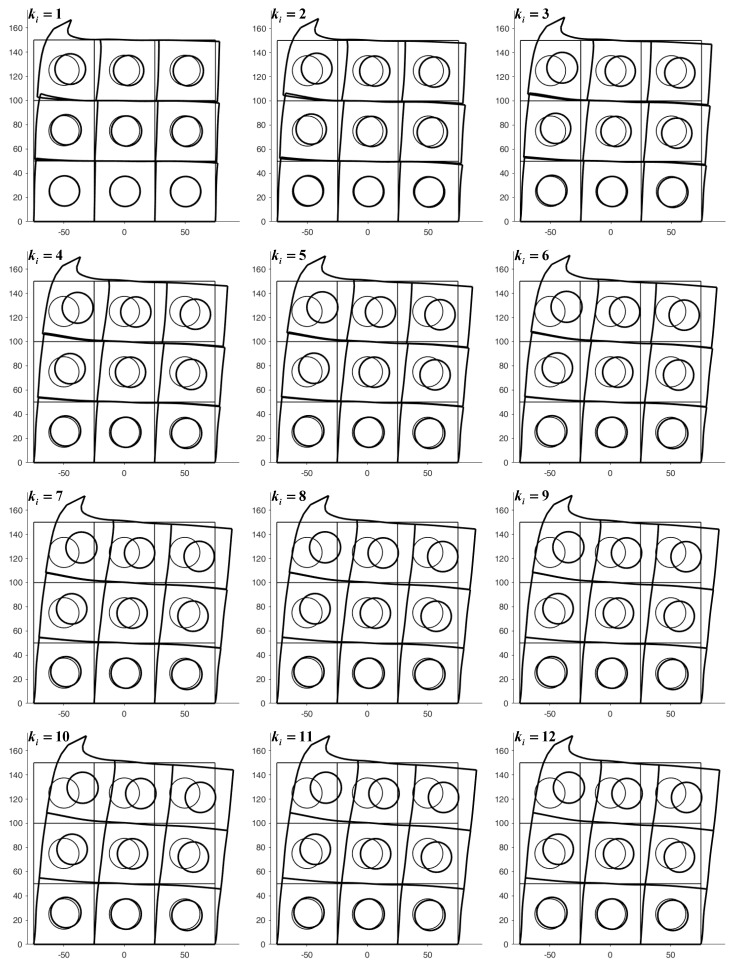
Stabilization cycle on the rows: deformed configurations provided by the $k_{i}\text{-}\mathrm{th}$ top-to-bottom-to-top iteration, for $1\leq k_{i}\leq 12$ (Thin line: undeformed configurations. Thick line: deformed configurations. Amplification factor of the displacements: k=200 ).

The square element of side L=50 mm in Figure 20 contains a round inclusion with radius R=25 mm, centered on the barycenter of the square element. The Young’s modulus and Poisson’s ratio of the matrix in Figure 20 are the same as in the example of Section 4.1 ($E=2\cdot 10^{7}N/m^{2}$,ν=0.3), while the mechanical properties of the round inclusion are $E=2\cdot 10^{10}N/m^{2}$, ν=0.3. Figure 21 shows the first 12 top-to-bottom-to-top iterations for a 3×3 array, generated by the discrete element with round inclusion in Figure 20.

Since the inclusion introduces a discontinuity in the material properties to improve the numerical solution, it is convenient to refine the mesh along the contour of the inclusion (Figure 20). The DECM uses an adaptive mesh generator that allows both the refinement and the coarsening of the mesh [59]. Furthermore, the mesh generator also provides a second mesh, linked to the first mesh by a relationship of geometric duality. In fact, as already pointed out in Section 3.1, the CM requires the generation of two meshes since it enforces the compatibility on the elements of the first mesh and the equilibrium on the elements of the second mesh.

The first mesh used by the CM is the triangular mesh of Delaunay (Figure 20). For the second mesh, there are various generation criteria, depending on the duality relation chosen to link the two meshes. One possibility is to use the circumcenters of the Delaunay mesh and the midpoints of the Delaunay sides to generate the nodes of the second mesh. This gives rise to the mesh of Voronoi (Figure 20a). Another possibility is to use the barycenters and midpoints of Delaunay (Figure 20b). The second possibility is preferable to the first one because, for very complex domain geometries, the mesh generator cannot guarantee that all Delaunay triangles are acute. This is a problem from the numerical point of view because the circumcenter of an obtuse triangle is outside the triangle, which causes a sign error for that triangle.

Since the main target of this work is to provide the first exploratory analysis on the ability of the DECM approach to model composite continua by using a series of discrete elements, the possible debonding phenomena at the interface between matrix and inclusions are outside the interests of this paper. Therefore, this section presents the results of the DECM static analysis in the assumption of a perfect interface between the matrix and inclusions, which leaves future studies on the DECM the task of dealing with interface debonding in composites. Nevertheless, as already pointed out in Section 4, the DECM uses the same numerical tools as the CM, even those developed for crack propagation. This means that the DECM can take into account the interface debonding by means of the failure criterion in the Mohr/Coulomb plane used to study tile detachments in radiant heat floors [63].

The presence of stiff inclusions does not cause stabilization problems in restoring the continuity between discrete elements. In fact, the code reaches the numerical convergence in almost the same number of iterations with respect to the case of absent inclusions.

**Figure 22:**
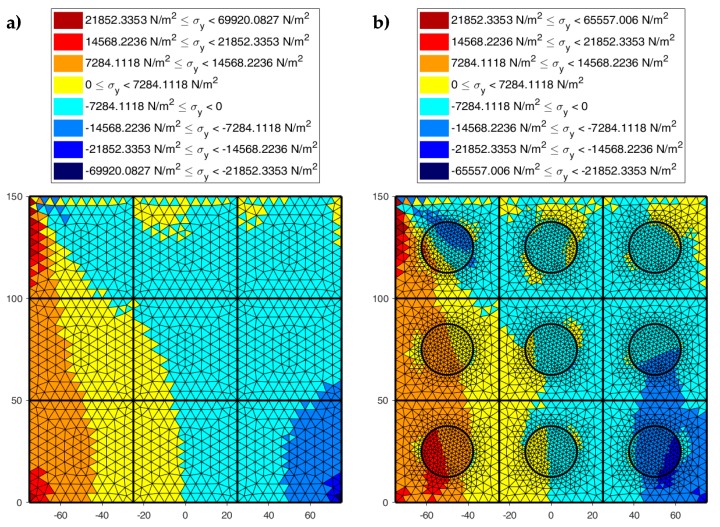
Normal stresses $\sigma_{y}$ (the y-axis runs vertically) in the discrete elements of a 3×3 array subjected to shear load: (**a**) Without inclusions and (**b**) with round inclusions, which are stiffer than the matrix.

**Figure 23:**
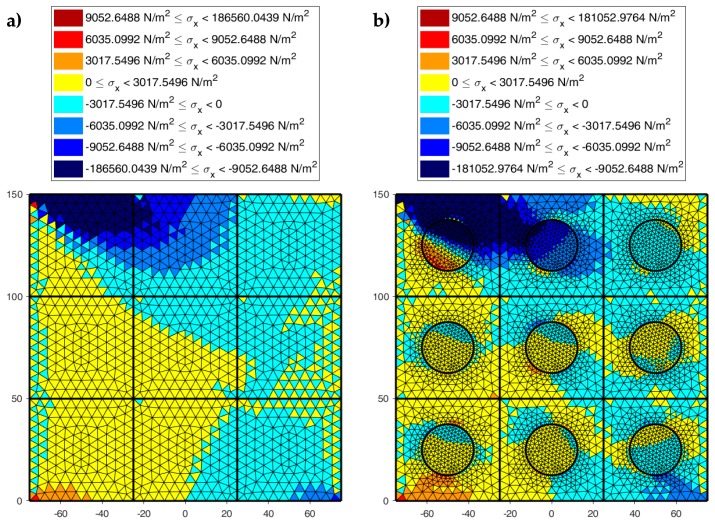
Normal stresses $\sigma_{x}$ (the x-axis runs horizontally) in the discrete elements of a 3×3 array subjected to shear load: (**a**) Without inclusions and (**b**) with round inclusions, which are stiffer than the matrix.

**Figure 24:**
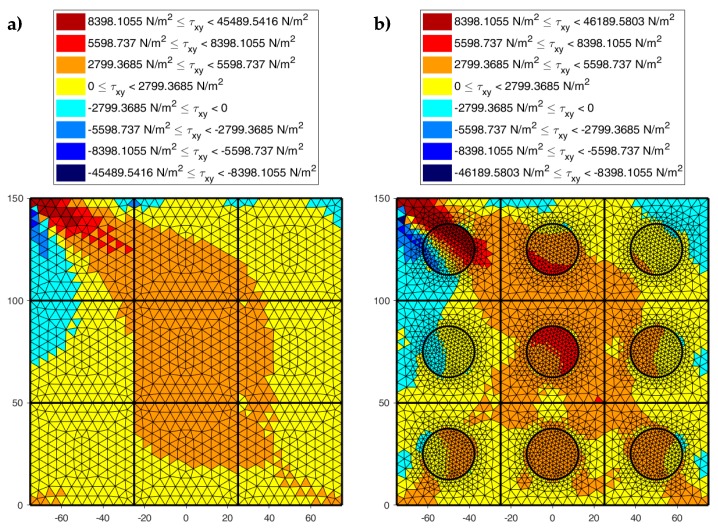
Shear stresses $\tau_{xy}$ in the discrete elements of a 3×3 array subjected to shear load: (**a**) Without inclusions and (**b**) with round inclusions, which are stiffer than the matrix.

Figure 22, Figure 23 and Figure 24 use a discrete color map to facilitate the comparison of stress values between the two-dimensional domains with and without inclusions. Furthermore, the color ranges are not equally wide, since the ranges of the maximum and minimum stresses are wider than the other ranges. This allows us to identify the areas in which, most likely, the material yields due to the point load. Since the elastic solution provided by the model is no longer valid in the yielding areas, the non-uniform subdivision of the color ranges focuses on the elastic areas.

As shown in Figure 22, Figure 23 and Figure 24, the stress field does not show evident discontinuities in crossing the boundaries of adjacent discrete elements. This means that, after restoring continuity, the set of individual discrete elements actually provides the same stress field obtained for the non-discretized two-dimensional domain. This is a remarkable result since the use of only local stiffness matrices without ever performing the static analysis on the assembled domain does not guarantee the continuity of the stress field in the whole domain.

**Figure 25:**
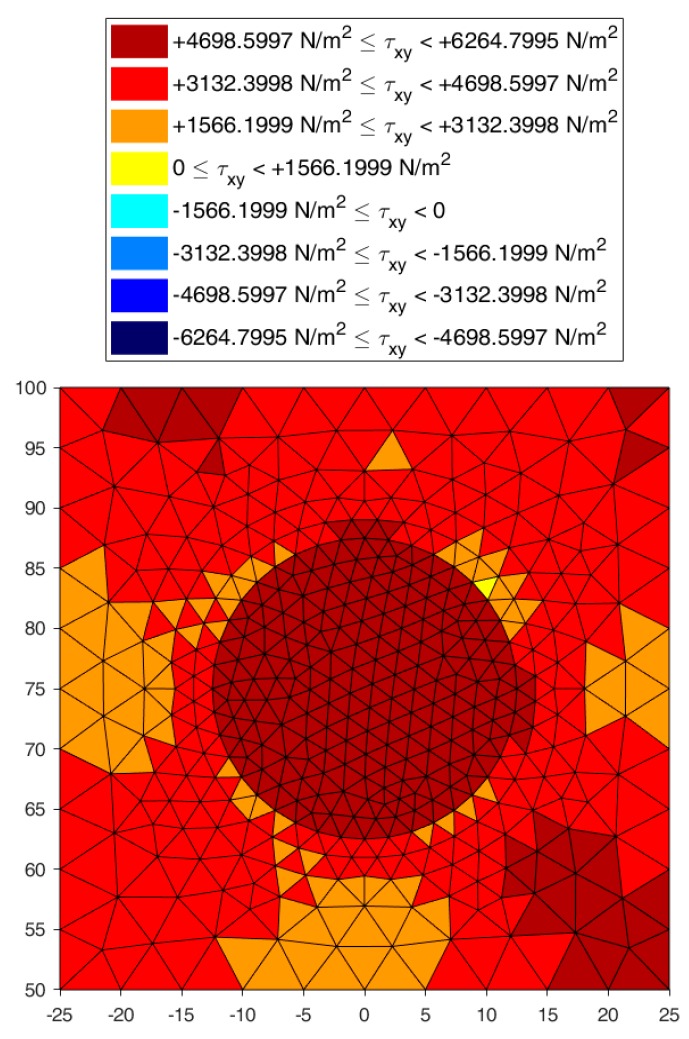
Detail of the shear stress $\tau_{xy}$ for the discrete element i=2, j=2 (colors equally ranged between the extreme values of shear stress for the element).

Figure 22, Figure 23 and Figure 24 also show how the inclusions modify the stress field. Due to the greater stiffness of the inclusions with respect to the matrix, the stresses tend to concentrate within the inclusions, which unloads the areas close to them. This is particularly evident in Figure 25, which shows the plot of the shear stress for the discrete element i=2, j=2 (second row and second column).

The high stress values reached within the inclusions can cause damage to the inclusions. In particular, the high values of shear stress can split the inclusions into two parts, which actually happens in many experimental shear tests. Furthermore, in Figure 22b, Figure 23b, and Figure 24b, the stresses of the inclusions tend to increase (in absolute value) near the boundaries of the inclusions.

This makes the interfaces between the matrix and inclusions particularly vulnerable and explains the interface detachments, which is often observed experimentally.

Lastly, Figure 22, Figure 23 and Figure 24 show that the inclusions reduce the maximum value of the normal stress in the matrix (for both $\sigma_{x}$ and $\sigma_{y}$), while, at the same time, increasing the maximum value of the shear stress, $\tau_{xy}$. Therefore, although the inclusions improve the strength of the composite material, they can increase the vulnerability to shear loads.

### 4.3. The Effect of the Inclusions for Axial Loads

#### 4.3.1. Comparison with the Results of a Previous CM Analysis

In the spirit of comparison between the DECM and the CM, we will now compare the results of the DECM model with those of a previous numerical analysis with the CM. Actually, as mentioned in Section 1, the parametric structure of the DECM code allows the operator to choose both the elastic properties and the geometric characteristics of the problem as well as the loading condition. In particular, the code asks the operator to define the following.

- Young’s modulus of both the inclusion and the matrix;
- Poisson’s ratio of both the inclusion and the matrix;
- Length of the base of the discrete element;
- Height of the discrete element (not necessarily equal to the base);
- Shape of the inclusion (no inclusion, round inclusion, polygonal inclusion with a randomly generated shape [107], or straight crack that form a random angle with the x-axis);
- Radius of the round inclusion, which also serves to generate the polygonal inclusion [107];
- Coordinates of the center of the inclusion;
- Number of subdivisions of the base (to generate the mesh);
- Number of subdivisions of the height (to generate the mesh);
- Number of subdivisions of the circular contour (in the case of round inclusion);
- Number of rows of the array;
- Number of columns of the array;
- Loading condition (concentrated force or distributed load);
- Position of the load (one of the two unconstrained corners or one of the midpoints of the three unconstrained sides, in the case of concentrated forces and one of the three unconstrained sides, in the case of distributed loads);
- Intensity of the load.

This allows a direct comparison between the DECM and previous CM analyses on composite continua consisting of a single element with one inclusion, which sets both the number of rows and the number of columns equal to 1.

**Figure 26:**
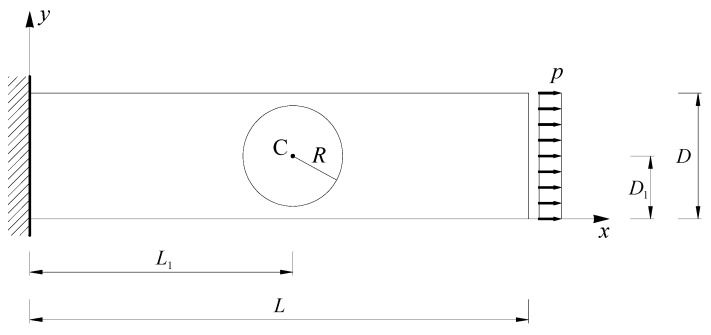
Geometry and loading condition of the elastic cantilever beam with a round inclusion [5].

The reference CM results are those obtained for an elastic cantilever beam with round inclusion [5] (Figure 26, geometric parameters of the CM model in Table 1). The elastic properties used for the matrix and the inclusion are the same as those of the element in Figure 20.

**Figure 27:**
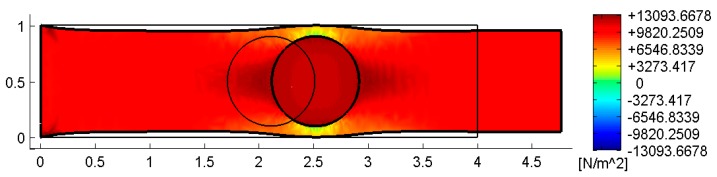
CM static analysis: normal stresses $\sigma_{x}$ plotted on the deformed configuration (Thin line: undeformed configuration. Thick line: deformed configuration. Amplification factor of the displacements: k=500 ) [5].

**Figure 28:**
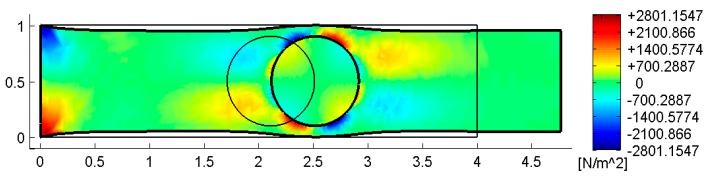
CM static analysis: shear stresses $\tau_{xy}$ plotted on the deformed configuration (Thin line: undeformed configuration. Thick line: deformed configuration. Amplification factor of the displacements: k=500 ) [5].

Figure 27 gives the axial stresses $\sigma_{x}$ identified for a tensile load $p=p_{x}=10\;\mathrm{kN}/m^{2}$, which is uniformly distributed along the right side [5]. Along the bi-material cross-sections in Figure 27, $\sigma_{x}$ increases where the local stiffness is higher and decreases where the local stiffness is lower. The CM numerical solution also provides non-zero values of $\tau_{xy}$, which are higher near to the left constraint and along the boundary of the inclusion, outside of inclusion (Figure 28).

The deformed configurations in Figure 27 and Figure 28 are consistent with the effect of the Poisson’s ratio on a bi-material. Since the Poisson’s ratio is the negative of the ratio of the (signed) transverse strain, $\varepsilon_{y}$, to the (signed) axial strain, $\varepsilon_{x}$, the transverse strain $\varepsilon_{y}$ is negative for uniaxial traction and reaches its maximum absolute value where $\varepsilon_{x}$ is at a maximum. Therefore, the shrinkage of the cross-section is greater for the higher values of $\varepsilon_{x}$, which, in turn, depends inversely on the local stiffness. In Figure 27 and Figure 28, the cross-sections actually shrink more where the local stiffness is lower, which is outside of inclusion.

The DECM analysis for the 1×1 array is, in all respects, identical to the CM analysis on a single element with one inclusion. In fact, since there is only one discrete element in the 1×1 array, the DECM code does not activate the iterative procedure described in Section 4.1 and performs a single static analysis with the CM.

**Figure 29:**
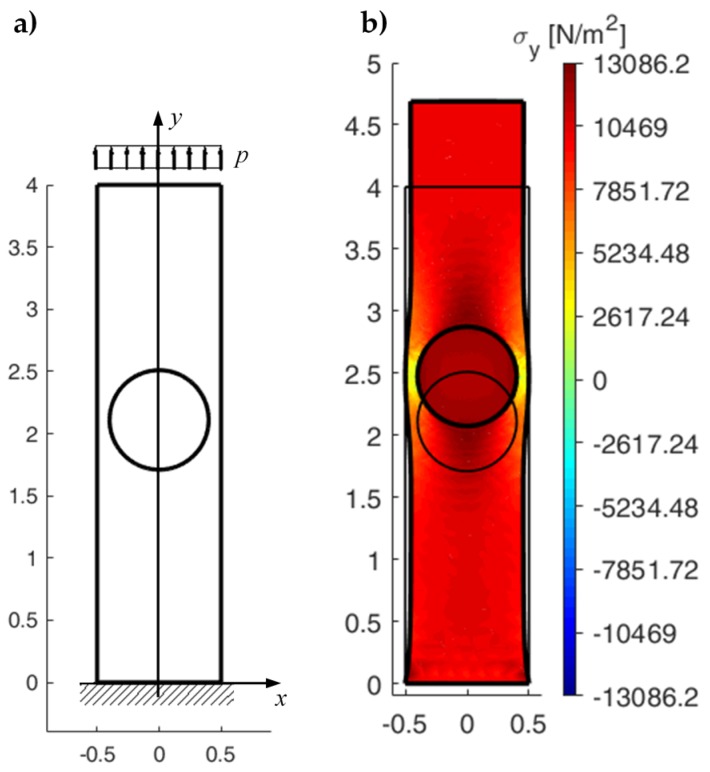
DECM static analysis for the 1×1 array generated by the discrete element with the geometric characteristics defined in Table 2. (**a**) Array geometry and loading conditions. (**b**) normal stresses $\sigma_{y}$ plotted on the deformed configuration (Thin line: undeformed configuration. Thick line: deformed configuration. Amplification factor of the displacements: k=500 ).

By changing the number of both the rows ($n_{r}$) and the columns ($n_{c}$) from 3 to 1 and choosing a load distributed on the upper side instead of a point load on the upper left corner, the DECM code modifies the 3×3 array of Section 4.2 into the 1×1 array that allows direct comparison between the DECM and CM analyses, after exchanging the *x* and *y* directions. In particular, for the geometric parameters in Table 2 and an axial load $p=p_{y}=10\;\mathrm{kN}/m^{2}$, the DECM code provides the geometric model in Figure 29a and the axial stresses $\sigma_{y}$ in Figure 29b. The maximum value of $\sigma_{y}$ in Figure 29b differs from the maximum value of $\sigma_{x}$ in Figure 27 by a factor of the order of $10^{-4}$ (percentage difference equal to 0.057%). This small difference depends on the two discretization meshes, which are slightly different in the shape despite the same number of subdivisions along the sides of the two discrete elements and along the two circular contours.

**Figure 30:**
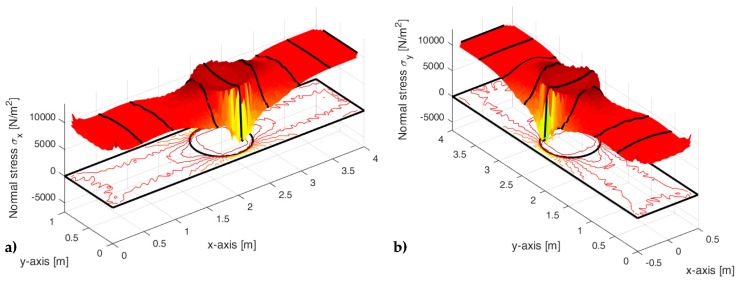
3D plots and isolines of the axial stresses for: (**a**) The numerical results of the CM model and (**b**) the numerical results of the DECM model for $n_{r}=1$ and $n_{c}=1$.

Furthermore, the axial stress reaches its maximum value on the constraint—due to the concentration of the stresses at the ends of the constraint—while the axial stresses on the loaded sides actually have the same value in the two cases ($10\;\mathrm{kN}/m^{2}$), as shown by the 3D plot of the axial stresses interpolated on a regular grid (Figure 30).

The use of a third axis in Figure 30a and Figure 30b allows us to appreciate the substantial equivalence between the two numerical solutions in more detail. It is worth noting that the lack of some data along the contours of the 3D plots in Figure 30 depends on the size of the regular grid used to interpolate the numerical solutions to build the 3D surfaces. Actually, as shown in Figure 27 and Figure 29b, both the CM and the DECM provide the stress values in the whole domain.

The thick line curves on the 3D surfaces in Figure 30 are the plots of the axial stresses at predetermined distances $d_{i}$ from the constraint, with i=1, 2,…, 8:

- $d_{1}=0.5\;m$ (stresses calculated along a line at a distance equal to 1/8 of the longest side);
- $d_{2}=1\;m$ (stresses calculated along a line at a distance equal to 1/4 of the longest side);
- $d_{3}=1.71\;m$ (stresses calculated along a line tangent to the inclusion, on the constraint side);
- $d_{4}=2.11\;m$ (stresses calculated along a line that passes through the center of the inclusion);
- $d_{5}=2.51\;m$ (stresses calculated along a line tangent to the inclusion, on the opposite side of the constraint);
- $d_{6}=3\;m$ (stresses calculated along a line at a distance equal to 3/4 of the longest side);
- $d_{7}=3.5\;m$ (stresses calculated along a line at a distance equal to 7/8 of the longest side);
- $d_{8}=4\;m$ (stresses calculated on the loaded side).

**Figure 31:**
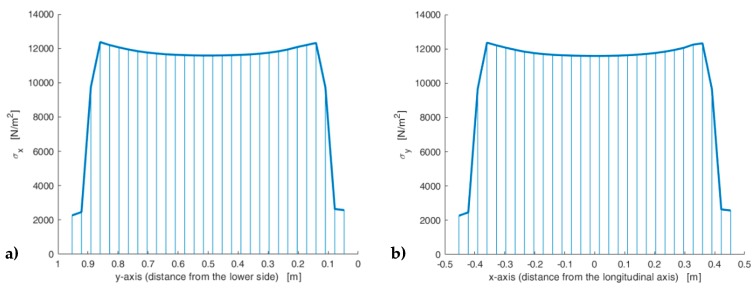
Axial stresses calculated at a constant distance from the constraint, along a line that passes through the center of the inclusion for: (**a**) The numerical results of the CM model and (**b**) the numerical results of the DECM model for $n_{r}=1$ and $n_{c}=1$.

In particular, Figure 31 shows the interpolation along the line that passes through the center of the inclusion ($d_{4}=2.11\;m$) of the axial stresses calculated by the CM and the DECM. As for Figure 30, the lack of data for the points on the contour depends on the interpolation grid.

#### 4.3.2. DECM Results for a Periodic Composite Specimen

**Figure 32:**
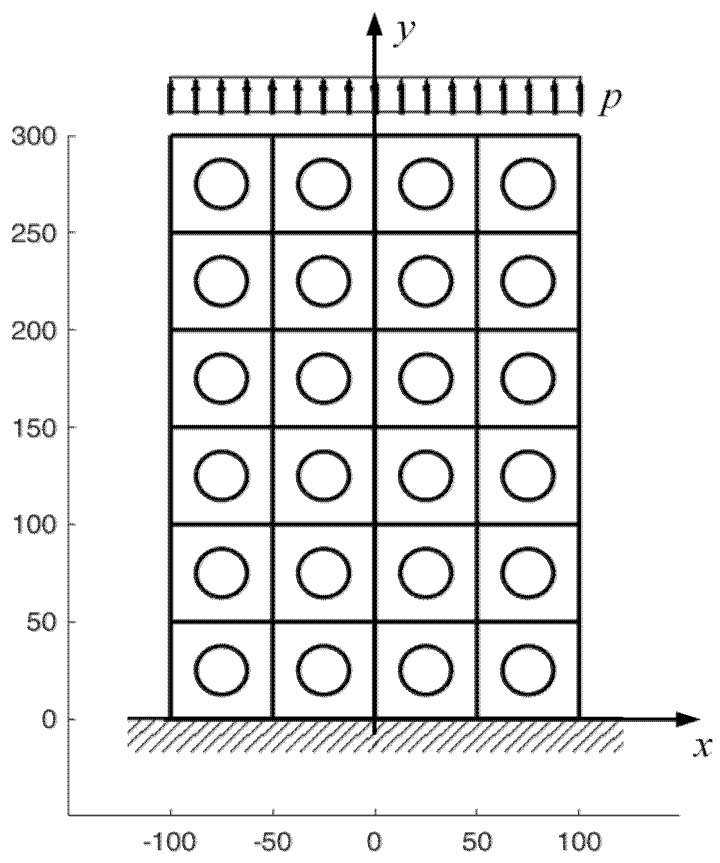
Geometry and loading condition of the 6×4 array with round inclusions (linear measurements in mm).

Let us now consider the 6×4 array generated by the discrete element with round inclusion of Figure 20 and subjected to a uniaxial traction $p=p_{y}=10\;\mathrm{kN}/m^{2}$ (Figure 32).

The 3/2 ratio between $n_{r}$ and $n_{c}$ allows the stress field of the central rows not to depend on the effect of the constraint and the stress distortion on the loaded side.

**Figure 33:**
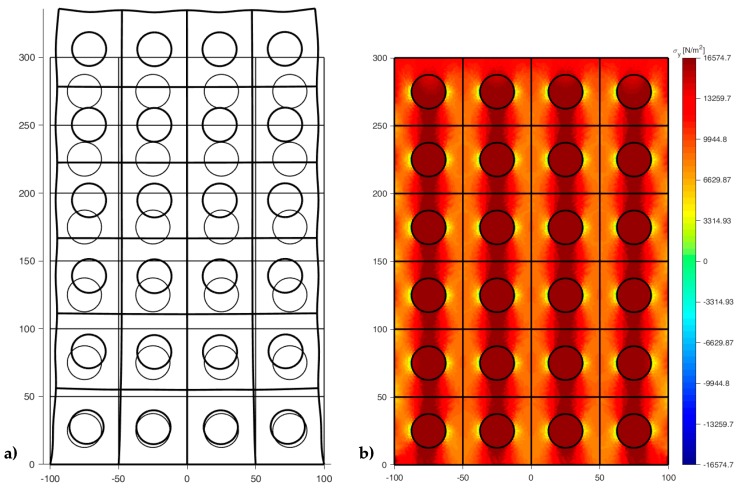
Elastic modeling of the 6×4 array subjected to uniaxial traction: (**a**) Deformed configuration (amplification factor of the displacements: k=400 ) and (**b**) normal stresses $\sigma_{y}$.

**Figure 34:**
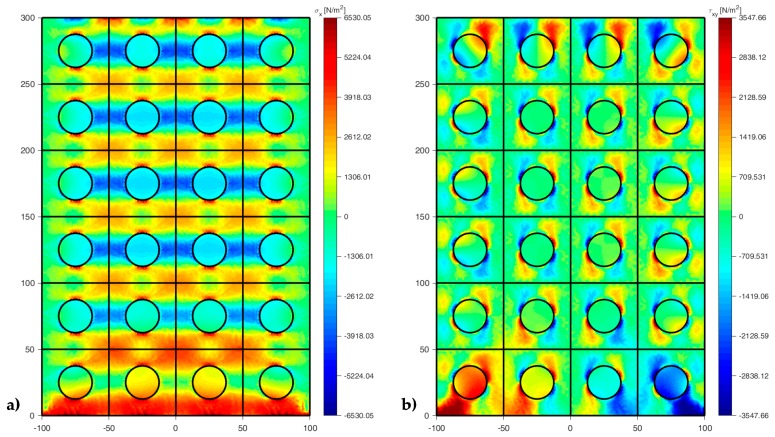
Stress fields in the 6×4 array subjected to uniaxial traction. (**a**) Normal stresses $\sigma_{x}$ and (**b**) shear stresses $\tau_{xy}$.

Figure 33 and Figure 34 show that the DECM is actually able to reproduce the same results obtained with the CM on a greater scale. In particular, Figure 33a provides the deformed configuration of the 6×4 array in Figure 32 by taking into account the Poisson effect and the reciprocal constraints between discrete elements. In fact, since the discrete elements prevent the adjacent elements of the same row from freely shrinking or expanding in the transverse direction, on the vertical inner sides in Figure 34a, the DECM code identifies positive and negative normal stresses $\sigma_{x}$ that would not be present in the continuum in the absence of inclusions. Moreover, the difference in stiffness between the matrix and the inclusions leads the normal stresses $\sigma_{x}$ to concentrate above and below the inclusions, close to the interfaces.

The numerical field of $\sigma_{x}$ in Figure 34a is symmetric with respect to the longitudinal axis, as it must be in order to comply with the equilibrium conditions in the direction of the x-axis (no horizontal force in the direction of the x-axis).

The difference in stiffness between the matrix and the inclusions is also the main cause of the shear stresses that arise along the boundaries of the inclusions (Figure 34b), which is already observed in the elastic cantilever beam (Figure 28). Actually, the elastic solution of De Saint Venant for a homogeneous material subjected to axial load returns zero values for both $\sigma_{x}$ and $\tau_{xy}$. The numerical field of the shear stresses in Figure 34b is skew-symmetric with respect to the longitudinal axis. This nullifies the resultant of the shear stress on each cross-section, as it must be in order to comply with the equilibrium conditions.

The concentration of stresses $\sigma_{x}$ and $\tau_{xy}$ near the constraint on the lower sides can damage the continuum and be the cause of an early crack initiation. Moreover, in Figure 34a,b, the constraint modifies the stress field up to a distance almost equal to half the length of the constraint (twice the side of the discrete elements).

In a real tensile test, this is the reason for using dog bone specimens, which have a shoulder at each end and a gauge section in the central part. The shoulders are wider than the gauge section to avoid early failure at the ends and ensure a greater probability that the sample breaks in the midsection.

**Figure 35:**
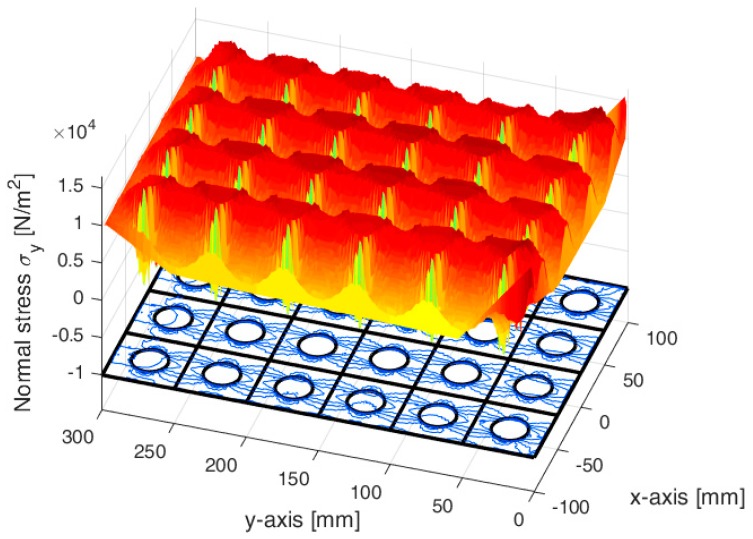
3D plot and isolines of the normal stresses $\sigma_{y}$.

The normal stresses $\sigma_{y}$ in Figure 33b follow the general behavior of the axial stresses in Figure 27. The low deformability of the inclusions leads the material enclosed between the inclusions of the same column to stretch more than the remaining areas of the matrix. As a result, these areas bear normal stresses $\sigma_{y}>p_{y}$, which are greater than the average axial stress of the matrix (Figure 35). The low deformability of the inclusions also leads the material to the right and left of the inclusions to stretch less than the remaining areas of the matrix. Consequently, these areas—in particular, those close to the inclusions—bear normal stresses $\sigma_{y}<p_{y}$, which are lower than the average axial stress of the matrix. This is the same effect, similar to the negative skin friction, already observed for brick masonry walls [52] (Section 2). Similar to what happens for the stresses $\sigma_{x}$, even the numerical field of the $\sigma_{y}$ is symmetric with respect to the longitudinal axis, in accordance with the equilibrium conditions.

Lastly, the 3D plot of $\sigma_{y}$ (Figure 35) allows us to appreciate the behavior of the axial stresses on the loaded sides (y=300 mm), where $\sigma_{y}$ is equal to the applied load: $\sigma_{y}=p_{y}=10\;\mathrm{kN}/m^{2}$.

**Figure 36:**
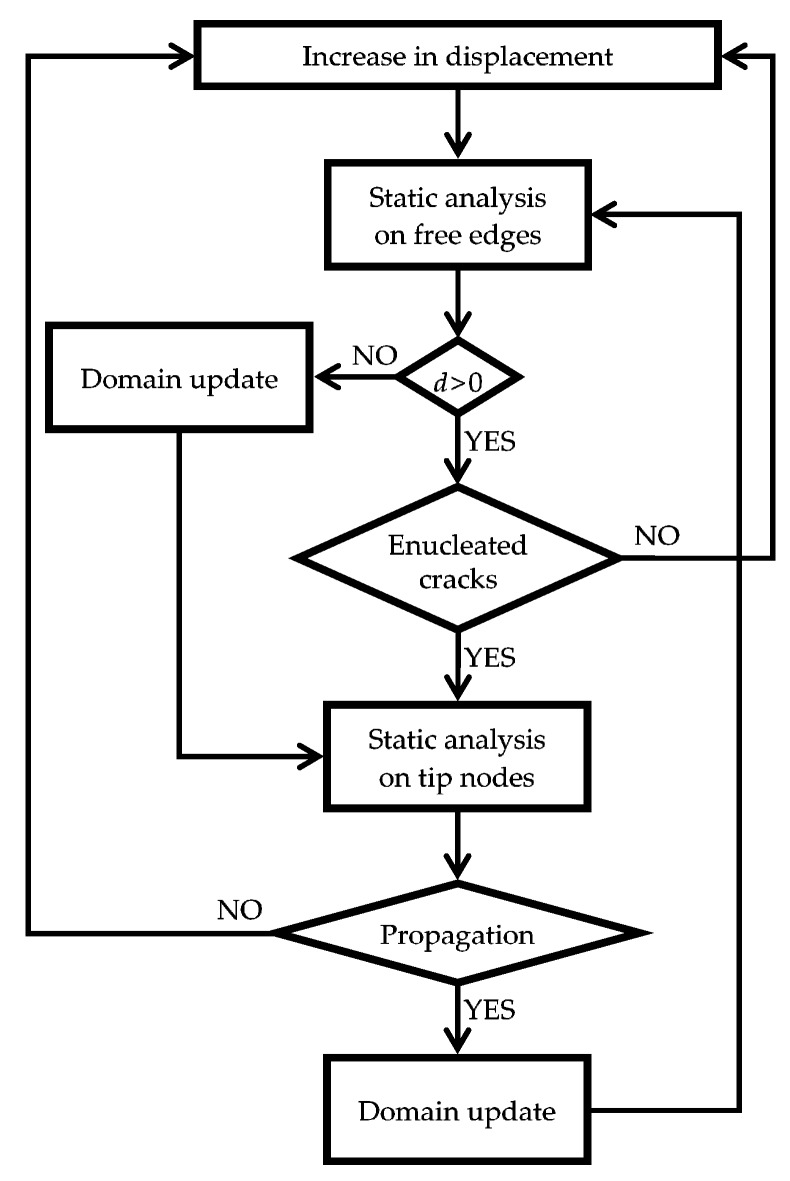
Flow chart of a CM code for the analysis of crack propagation in the displacement control.

The combination of stresses $\sigma_{x}$ and $\sigma_{y}$ above and below the inclusions makes the interfaces between matrix and inclusions particularly vulnerable. Therefore, the upper and lower interfaces are, most likely, sites of enucleation of the cracks, which originate as disconnections between matrix and inclusions. As already pointed out, after crack enucleation, the DECM code can estimate the propagation direction as the CM, in the Mohr/Coulomb plane (Section 3.2). In particular, Figure 36 shows the flow chart of a CM code in the displacement control, where d is the distance between the largest Mohr’s circle and the limit domain of Leon (when d<0, the circle intersects the limit domain, which means that the material has reached its maximum strength).

## 5. Future Developments

Further improvements of the DECM code presented in this paper consist of the analysis of the stress field for composite continua with randomly shaped inclusions [107] and/or randomly distributed inclusions. The code structure also allows the substitution of the solid inclusions with straight cracks that form a random angle with the x-axis. Both the solid inclusions and the straight crack can cause local disconnections, which propagate through the composite continuum. Considering crack propagation is not a problem with the DECM, as it uses the tools already developed for the CM (Section 3.2). Furthermore, regardless of the shape of the inclusions and the possible presence of cracks that propagate, the storage of the stiffness matrices in separate workspaces allows different processors to perform the static analysis on different elements simultaneously. The most appropriate parallelization technique for the DECM is still under evaluation [108]. However, in the author’s opinion, it is reasonable to assume that the advantage of the parallelization will be proportional to the number of discrete elements used to model the continuum since this number is equal to the number of required workspaces and, consequently, of processors that can run the numerical analyses simultaneously.

**Figure 37:**
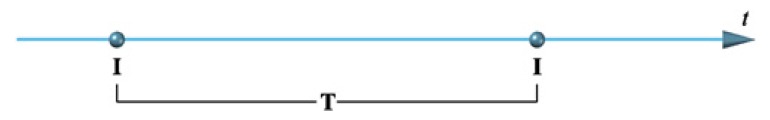
The two-time elements of the CM, represented as elements of a one-dimensional cell-complex.

Lastly, the static solution of the DECM allows an easy extension to dynamic problems. Actually, the CM is also useful for solving problems in the time domain, using the same explicit finite difference solution scheme of the DEM. In fact, the CM associates also the global variables in time with the elements of a cell-complex, which has a dimension of 1 and generalizes the time-axis (Figure 37). The time elements of the CM are two: the time instant, I, and the time interval, T. They are the nodes and sides, respectively, of the one-dimensional cell-complex that represents time (Figure 37). According to the nomenclature of algebraic topology, the time instants are the boundaries, or the faces, of the time intervals.

**Figure 38:**
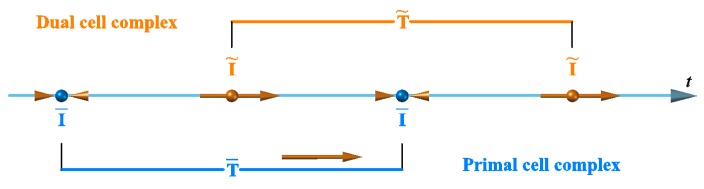
Time elements in the primal and dual cell-complexes.

Even for the time elements, the CM uses a second cell-complex (the dual cell-complex), linked to the first cell-complex (the primal cell-complex) by a relationship of geometric duality. In one-dimensional spaces, the dual (orthogonal complement) of a point is a line segment and the dual of a line segment is a point. Therefore, the nodes of the dual cell-complex are the middle points of the primal sides (Figure 38). Consequently, the two cell-complexes in time are staggered of τ/2, where τ is the constant time step. The geometric duality between the elements of the two cell-complexes in time extends to their orientations (Figure 38).

A dual time interval, $\tilde{T}$, is to the enclosed primal instant, $\bar{I}$, as an influence region is to its inner node. Analogously, the primal time interval, $\bar{T}$, is the influence region of its dual instant, $\tilde{I}$.

The CM computes the displacements at instants $\bar{I}$ of the primal cell complex. Let ${\bar{u}}_{h}^{n}$ and ${\bar{u}}_{h}^{n+1}$ be the displacements of node h at instants ${\bar{I}}_{n}$ and ${\bar{I}}_{n+1}$, respectively. Then, the velocity ${\tilde{v}}_{h}^{n+1/2}$ and the linear momentum ${\tilde{p}}_{h}^{n+1/2}$ of node h in the time interval ${\bar{T}}_{n+1/2}$ of boundaries ${\bar{I}}_{n}$ and ${\bar{I}}_{n+1}$ are associated with the dual instant ${\tilde{I}}_{n+1/2}$.

(7) $${\tilde{v}}_{h}^{n+1/2}=\frac{1}{\tau }\left({\bar{u}}_{h}^{n+1}-{\bar{u}}_{h}^{n}\right),$$

(8) $${\tilde{p}}_{h}^{n+1/2}=\frac{m_{h}}{\tau }\left({\bar{u}}_{h}^{n+1}-{\bar{u}}_{h}^{n}\right),$$

where $m_{h}$ is the mass of node h.

Lastly, the total force acting on node h in the dual time interval ${\tilde{T}}_{n}$ of boundaries ${\tilde{I}}_{n-1/2}$ and ${\tilde{I}}_{n+1/2}$ is associated with the primal instant ${\bar{I}}_{n}$.

(9) $${\bar{T}}_{h}^{n}+{\bar{F}}_{h}^{n}=\frac{1}{\tau }\left({\tilde{p}}_{h}^{n+1/2}-{\tilde{p}}_{h}^{n-1/2}\right),$$

where ${\bar{T}}_{h}^{n}$ and ${\bar{F}}_{h}^{n}$ are the surface and volume forces acting on node h.

## 6. Conclusions

This paper describes the structure of the DECM, which is a multiscale numerical method, introduced in this paper for the first time and specifically dedicated to the modeling of periodic composite continua. The new numerical method stems from the need to extend an accurate description provided on the micro-scale by the Cell Method (CM) to the macro-scale. The key idea of the DECM is to reduce the typical computation time of a macro-scale analysis with the CM by subdividing the domain into sub-domains and performing the static analysis on each sub-domain (on a lower scale) independently of the other sub-domains. This is a revolutionary idea for the CM, as it is the first attempt to extend the CM to the macro-scale by performing only local analyses with few degrees of freedom without having to use a global stiffness matrix for the whole domain. The result is an approach with distinct discrete elements, which connotes the new numerical method as a DEM in the broad sense, as it conforms to the original definition of DEM proposed by Cundall and Hart.

The set of discrete elements reproduce the continuum. Therefore, the DECM does not require filling a geometric form with particles to reproduce the shape of the continuum. This allows us to avoid one of the most delicate DEM phases, namely, the generation of the model geometry, which also involves time-consuming calibrations and scaling processes.

Since the boundary conditions on the sub-domains are unknown at the beginning of the computation, the DECM code identifies them through stabilization cycles on the displacements, which restore the continuity between the discrete elements. The stabilization algorithm arranges the discrete elements into ordered arrays, which improves the efficiency of the algorithm while keeping computation times low.

The most important advantage of the DECM concerns the use of the CM, which—being an algebraic approach—overcomes some of the typical drawbacks of the differential formulation. Specifically speaking, the CM can easily treat any type of singularity, including concentrated forces and discontinuities in the rheological properties. Therefore, unlike the Finite Element Method (FEM), the CM provides descriptions up to the scale of single inclusions and interfaces between different materials. This is precisely the main reason to use the CM on the micro-scale, leaving the DECM to manage the discrete elements in order to capture the behavior on the macro-scale.

Another unique feature of the CM is its intrinsic nonlocality, which means that the global variables naturally include the length scales. This means that, unlike the DEM, the DECM does not require particular contact constitutive relations to establish interactions between the aggregates that are not in direct contact, when they are within a predetermined interaction range. In fact, the nonlocal nature of the variables allows the DECM to take into account the medium-range and long-range interactions automatically.

Compared to other DEM methods, the DECM does not obtain the static solution as a steady state solution of a dynamic problem, but performs the computation directly in the space domain. This entails two other advantages from the numerical point of view:

- The DECM does not require the calibration of the stable time step, which is, instead, needed by the DEM to allow the convergence of the numerical solution;
- The DECM does not require a preliminary assessment of the minimum number of contact points to obtain the correct solution, which happens in the case of the DEM with deformable discrete elements to limit the computational cost of the dynamic relaxation technique.

Furthermore, unlike the DEM approaches with deformable blocks, the DECM has an internal mesh with satisfactory performance. Therefore, the DECM is useful for studying the stress field within deformable blocks and, in particular, within deformable blocks with inclusions of different materials.

To allow parametric analyses, the DECM code presented in this paper has a fully parametric structure, which means that it lets the user define the geometry of the discrete elements of the inclusion and of the array, as well as the elastic properties of the bi-material composite and the type of load. As an example, this paper provides the results of some DECM numerical analyses for shear and axial loads on periodic composite continua with round inclusions, which are stiffer than the matrix.

The numerical results highlight how a difference in the deformability causes adjacent bodies to interact with each other in terms of both displacements and stresses. In fact, due to the difference in the deformability, bodies consisting of different materials interact with a reciprocal degree of constraint on the displacements, which gives rise to stress components not explained by the elastic solution of De Saint Venant for homogeneous materials.

Moreover, the DECM analysis shows that the stresses concentrate within the inclusions of the periodic composite continua, which modifies the state of stress with respect to the case without inclusions for both loading conditions. Lastly, the DECM identifies some critical stress states close to the boundaries of the inclusions. In particular, the critical stress states are located within the inclusions for the shear load and outside the inclusions for the axial load.

The stress concentration within the inclusions can split the inclusions while the critical stress state along the boundaries of the inclusions can detach the inclusions from the matrix. The experimental tests for shear and axial loads provide extensive descriptions of both splitting and detachment of the inclusions in cementitious continua with aggregates. These failure mechanisms now find a numerical justification.

Like the CM codes, the DECM code is able to identify the points of enucleation of the cracks and simultaneously manage the propagations of different cracks, even in the event of crack bifurcation. Therefore, it is not necessary to know in advance the positions of crack enucleation, which is a further feature that distinguishes the DECM from the DEM codes for the analysis of composite continua and, specifically, from those DEM codes that model traditional URM (unreinforced masonry) buildings with rigid blocks. The DECM analysis of the pattern of crack propagation in periodic composite continua will be the subject of future work.

## Figures and Tables

**Table 1:** Geometric parameters used to model the beam of Figure 26 [5].

Symbol	Description	Value
L	Base	4 m
D	Height	1 m
R	Radius of the round inclusion	0.4 m
$L_{1}$	Distance of the center C from the left side	2.11 m
$D_{1}$	Distance of the center C from the lower side	0.5 m
$n_{div}^{L}$	Number of subdivisions of the base	32
$n_{div}^{D}$	Number of subdivisions of the height	8
$n_{div}^{crf}$	Number of subdivisions of the circular contour	80

**Table 2:** Geometric parameters of the discrete element used in the DECM analysis.

Symbol	Description	Value
L	Base	1 m
D	Height	4 m
R	Radius of the round inclusion	0.4 m
$L_{1}$	Distance of the center of the inclusion from the lower side	2.11 m
$D_{1}$	Distance of the center of the inclusion from the left side	0.5 m
$n_{div}^{L}$	Number of subdivisions of the base	8
$n_{div}^{D}$	Number of subdivisions of the height	32
$n_{div}^{crf}$	Number of subdivisions of the circular contour	80
